# Impacts of climate change on the livestock food supply chain; a review of the evidence

**DOI:** 10.1016/j.gfs.2020.100488

**Published:** 2021-03

**Authors:** C.M. Godde, D. Mason-D’Croz, D.E. Mayberry, P.K. Thornton, M. Herrero

**Affiliations:** a Commonwealth Scientific and Industrial Research Organisation, Agriculture and Food, St Lucia, QLD, 4067, Australia; b CGIAR Research Programme on Climate Change, Agriculture and Food Security (CCAFS), ILRI, Nairobi, Kenya

**Keywords:** Livestock, Climate change, Supply chain, Heat stress, Vulnerability, Risk

## Abstract

The potential impacts of climate change on current livestock systems worldwide are a major concern, and yet the topic is covered to a limited extent in global reports such as the ones produced by the Intergovernmental Panel on Climate Change. In this article, we review the risk of climate-related impacts along the land-based livestock food supply chain. Although a quantification of the net impacts of climate change on the livestock sector is beyond the reach of our current understanding, there is strong evidence that there will be impacts throughout the supply chain, from farm production to processing operations, storage, transport, retailing and human consumption. The risks of climate-related impacts are highly context-specific but expected to be higher in environments that are already hot and have limited socio-economic and institutional resources for adaptation. Large uncertainties remain as to climate futures and the exposure and responses of the interlinked human and natural systems to climatic changes over time. Consequently, adaptation choices will need to account for a wide range of possible futures, including those with low probability but large consequences.

## Highlights

• Risk results from the interaction of climate-related hazards with the exposure and vulnerability of human and natural systems.
• Climate change will impact the livestock sector throughout the food supply chain⁠—from farm production to human consumption.
• Key hazards relate to climate change trends but also, and importantly, to climate variability and climate extremes.
• Large uncertainties remain as to climate futures and the exposure and responses of the interlinked human and natural systems.
• Adaptation choices will need to account for a wide range of possible futures.

## Graphical abstract

**Figure:**
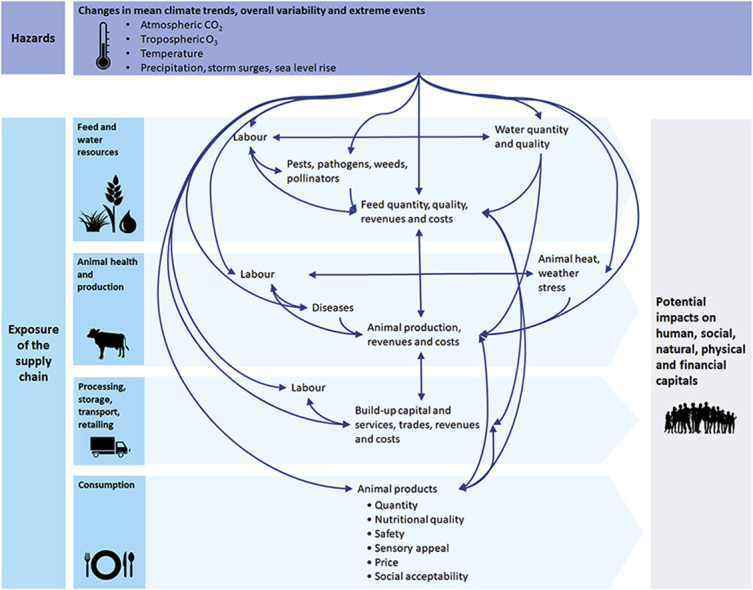

## 1 Introduction

Climate change is a major concern for current livestock systems worldwide. Global warming and its associated changes in mean climate variables and climate variability affect feed and water resources as well as animal health and production. Climate change also has implications for the processing, storage, transport, retailing and consumption of livestock products. The ability of current livestock systems to support livelihoods and meet the increasing demand for livestock products is thus threatened.

The livestock sector currently plays a key role in food supply and food security. Livestock products (meat, milk and eggs) contribute 15% and 31% of global per capita calorie and protein supply, with regional variations (FAOSTAT, 2020; see Appendix for calculation of estimates presented in the Introduction). About 30% and 6% of global ruminant meat and milk production originates from grazing systems, on land that is often poorly suited for cropping (Herrero et al., 2013). Furthermore, livestock provides a range of other services, including as a source of draught power, a means of transportation, a source of nutrients for poor soils, a source of income generation and diversification, and a form of financial capital, all of which contribute to the overall well-being and resilience of many communities (CIRAD, 2016). Over 844 million people worldwide receive some income from agriculture, and the livestock sector contributes about 40% of agricultural value-added (FAOSTAT, 2020; The World Bank, 2020a). Livestock contributions to food security and other sustainability dimensions will be affected by climate change, although the full extent and magnitude of the impacts remain unknown.

Livestock and climate change studies often focus on the climate mitigation potential of livestock and in describing adaptation practices. When studies cover climate impacts, these tend to have a relatively narrow viewpoint, focusing on specific livestock species, primary production, or on selected dimensions of risk of climate-related impacts such as climate hazards without considering vulnerability levels of different communities (e.g. of reviews, Escarcha et al. (2018); IPCC (2014); Rivera-Ferre et al. (2016); Rojas-Downing et al. (2017); Thornton et al. (2009)). In large part this reflects the fact that, compared to crop production, considerably less work has been published on observed and modelled climate impacts on livestock (IPCC, 2014a). It also reflects the limited number of synthetic reviews of the issue, as highlighted in Rivera-Ferre et al. (2016).

In order to fill this gap, we review the risk of climate-related impacts along the land-based livestock food supply chain (i.e. from production to consumption). While not exhaustive, we aim to capture the major trends with direct implications for livestock-sourced food availability, access, utilisation and stability, and highlight key recent literature. We acknowledge that the implications of climate change go well beyond these pillars and affect the provision of goods and services (e.g. wool, hides, skins and manure, animal traction, financial instrument, etc.), human livelihoods and health, ecosystems, economies, cultures, and infrastructure in complex ways. Also, while we recognize that climate adaptation strategies and the impacts of livestock on climate change are significant considerations, these are not covered here but assessed elsewhere (e.g. FAO (2018a), Escarcha et al. (2018), Henry et al. (2018), Herrero et al. (2016), Rivera-Ferre et al. (2016), Salman et al. (2019), Sejian et al. (2015) and Weindl et al. (2015)).

**Fig. 1:**
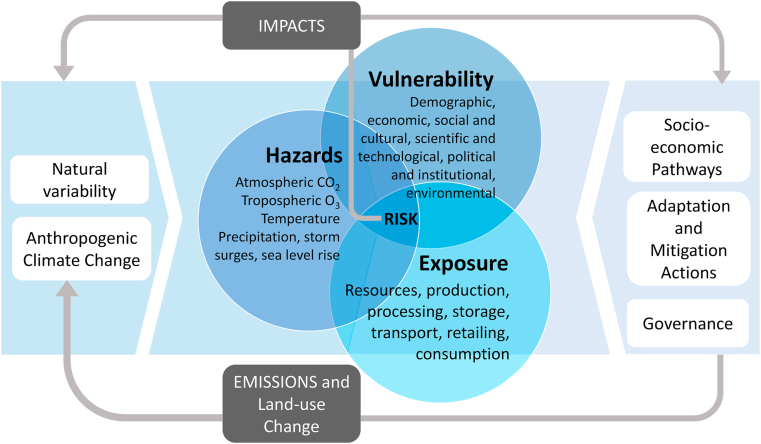
Schematic of the interaction among the physical climate system, exposure, and vulnerability producing risk in the livestock supply chain. Risk of climate-related impacts results from the interaction of climate-related hazards (including hazardous events and trends) with the vulnerability and exposure of human and natural systems. Changes in both the climate system (left) and socioeconomic processes including adaptation and mitigation (right) are drivers of hazards, exposure, and vulnerability. Adapted from IPCC (2014).

This review is framed around the concept of risk of climate-related impacts, as defined by the Intergovernmental Panel on Climate Change (IPCC) Working Group II (IPCC, 2014a). Risk of climate-related impacts results from the interaction of climate-related hazards with the exposure and vulnerability of human and natural systems (Fig. 1). The analysis of this interaction represents the core of the IPCC climate impacts assessments. We use the term hazard to refer to climate-related physical events or trends that impact livestock systems (IPCC 2014). Exposure refers to the parts of the livestock supply chain that could be adversely affected, while vulnerability encompasses humans’ capacity to cope and adapt to changes. The term impact is used primarily to refer to the effects of extreme weather, climate events and climate change on natural and human systems.

We first detail the extent to which the livestock supply chain is exposed to climate change, referring to key literature on topics that have received significant past attention and expanding on recent topics of concern. We then discuss the societal ability of the livestock sector to cope or adapt to changes considering broader societal trends before highlighting potential risks of climate-related impacts.

## 2 Exposure of human and natural systems

Anthropogenic greenhouse gas emissions are associated with a range of climate and atmospheric shifts, the impacts of which are already being observed (FAO, IFAD, UNICEF, 2018). Major trends that can impact the livestock food supply chain are increases in atmospheric carbon dioxide (eCO2) and tropospheric ozone (O3) concentrations; changes in both mean and variability of temperature and precipitation; sea level rise and storm surges; and increased risk and frequency of extreme weather events. Past and projected changes in these climate variables are detailed in the Supplementary Information.

**Fig. 2:**
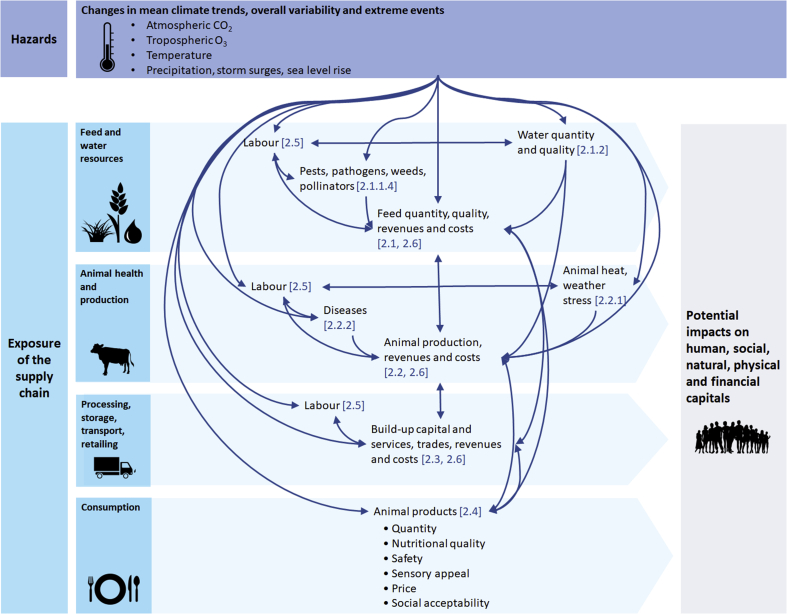
Potential impacts of climate-related hazards on the livestock land-based food supply chain. The term quantity encompasses here the notions of physical availability of feed and animal products, economic and physical access and stability of these products (availability, access and stability indicators as defined by FAO (2019)). The human livelihood capitals listed follow the sustainable livelihoods framework introduced by Scoones (1998) and thereafter modified (Ellis, 2000). Relevant text sections are provided in brackets.

These climate change hazards may adversely affect the livestock sector at different stages of the livestock supply chain, as summarised in Fig. 2 and Table 1, and further detailed in the sections below. The potential impacts on labour and prices and overall social dynamics, which affect all stages of the supply chain, are described last.

**Table 1:** Summary of the potential impacts of climate variables on the livestock food supply chain. The nature, extent and magnitude of the impacts will vary depending on a range of factors not described here that may interact in complex ways, such as interactions between climate variables, plant and animal species, agro-ecological conditions and socio-economic contexts.

Supply chain	Potential impacts of climate change
**Feed resources**	**Productivity** Regions already water stressed are likely to experience the most negative impacts. Some regions in high latitudes could experience yield increases due to reduced cold stress and longer growing seasons. Soil salinity in coastal regions may increase due to sea level rise and increased frequency and intensity of storm surges. Changing precipitation patterns particularly in arid regions could contribute to greater salinity. Changing weather patterns and warming temperatures could contribute to shifting pest and disease distribution and could increase stress on key pollinator species. Hotter and more humid conditions are likely to result in increased on-farm post-harvest losses where storage conditions are inadequate. Elevated eCO_2_ can increase yields but won't benefit all crops equally. Temperate C_3_ species could be the most positively affected, and realised benefits may be mitigated by water and nutrient constraints. Elevated O_3_ will have a negative effect on yields. **Nutritional quality** Warmer temperatures and drier conditions will tend to favour C_4_ species and increase toxicity in some plants, including during storage. Elevated eCO_2_ could reduce plant protein and mineral concentrations and increase toxicity in some species. Increases in eCO_2_ will tend to favour C_3_ plants and woody encroachment at the expense of grasses. **Variability in feed availability** Inter-annual climate variability is projected to increase globally with overall negative impact on feed production. Changes in seasonal climate patterns will have context specific impacts, which may be positive or negative. However, increased variability will likely lead to less predictable feed supply. Extreme events could restrict animal access to pastures and create larger disruptions to feed production.
**Water resources**	Hotter and drier conditions are likely to increase water requirements of plants and animals, increasing pressure on water resources, especially in regions already water stressed. Further, warming temperatures will contribute to greater glacier depletion disrupting historical surface water flows. Higher temperatures and extreme events such as floods and droughts are likely to decrease water quality for animal consumption, through increased concentration of pathogens, sediments, salts, nutrients or pollutants in water.
**Animal health and production**	Animal production, welfare and life expectancy are likely to be negatively impacted, through decreased feed availability and quality, heat stress, diseases (from outbreaks and weakened animal immune system) and mortality from extreme climate events such as storms, floods, heat and cold waves. Globally, the effects are likely to be negative, but in some geographies with cold winters, warmer temperatures may reduce animal cold stress and maintenance energy requirements, as well as housing heating.
**Processing, storage, transport and retailing**	Higher temperatures, increased humidity, increased frequency of extreme weather events, and rising sea levels are likely to put additional stress on built-up capital (machinery, transportation infrastructure, electricity networks, telecommunications, etc.). Further, warmer temperatures could increase the risk of animal heat stress during transportation, as well as worsen conditions for storage and distribution of food and feed, which could lead to reduced food quality, safety and shelf-life. Increased variability in production and extreme climate events will likely make trade patterns less regular, increasing reliance on complex logistic systems.
**Livestock products consumption**	Climate change can reduce the availability of livestock products, as well as their quality and safety through contamination with pathogens or pesticide and reduced nutritional quality and sensory appeal. Prices may increase and be more volatile. Changing social norms may impact diets, especially in high-income countries.
**Labour**	Labour availability and productivity is likely to be negatively impacted by climate change due to heat stress, increased risk of novel disease outbreaks, and extreme events like heat waves, floods and severe storms. Labour is also likely to be negatively impacted by exposure to decreased air quality associated with rising temperatures, nutrition from changes in food supply.
**Prices**	Costs along the supply chain, commodity price and price volatility are likely to increase under climate change. The impacts of climate change on animal product prices could be felt mainly through changes in costs and availability of feed.

### 2.1 Feed and water resources

#### 2.1.1 Quantity and quality of livestock feed production

Changes to the quantity and quality of livestock feed will be influenced by complex local interactions between eCO_2_ concentrations, tropospheric O_3_ levels, temperature, and precipitation. We first provide an inventory of how eCO_2_, O_3_, temperature and precipitation can affect livestock feed, then present some model projections under climate change. Livestock consume grains (especially in poultry, pig and intensive ruminant systems), crop above-ground biomass (e.g. in dual purpose crops which are both grazed and harvested), crop residues (e.g., straw or stover – key feed in mixed crop-livestock systems) as well as native and sown pastures (key feed in mixed crop-livestock and grazing-only systems). While not covered here, livestock can also be fed by-products and waste (e.g. oilseed cakes, bran, vegetable waste, brewer waste), concentrates and supplements (FAO, 2017).

##### 2.1.1.1 Direct impacts of atmospheric CO_2_ and tropospheric O_3_ on feed

Research shows that eCO_2_ may have both positive and negative impacts on livestock feed, although there is recent evidence that the fertilisation effects of eCO_2_ and nitrogen on plant physiological processes may slow in the future as ecosystems productivity become dominated by the negative effects of higher temperatures and extreme droughts (Peñuelas et al., 2017).

Increases in eCO_2_ concentrations stimulate plant primary productivity (see review in Ainsworth et al., 2020), increasing potential yields of some species. Plants with a C_3_ photosynthetic pathway such as wheat, rice, soybean and temperate grasses experience greater growth stimulation than C_4_ plants such as maize, sorghum, sugarcane and tropical grasses. However, the CO_2_ fertilisation effects can also reduce animal feed quality (Augustine et al., 2018; Myers et al., 2014; Smith and Myers, 2018). For example, Myers et al. (2014) reported that C_3_ crops other than legumes had lower grain protein concentrations under elevated eCO_2_ concentration in the range 546–586 ppm (−6.3% in wheat grains and −7.5% in rice grains). The impact on C_4_ crop grain was smaller. Increased eCO_2_ was also found to decrease the overall mineral concentrations (−8%) and increased the total non-structural carbohydrate (mainly starch, sugars) to mineral ratios in the total biomass of non-leguminous C_3_ plants (Loladze, 2014). While the nutritional quality of C_3_ grasses may be the most greatly impacted by eCO_2_ increases, it may nonetheless remain higher than C_4_ grasses under elevated eCO_2_ (Barbehenn et al., 2004). Increased toxicity has also been reported in some plants, with Gleadow et al. (2009) measuring a 160% increase in the concentration of cyanogenic glycosides (compounds that break down to release toxic hydrogen cyanide when plant tissue is crushed or chewed) in cassava leaves between CO_2_ concentrations of 360 and 710 ppm in greenhouse experiments. Woody encroachment associated with rising eCO_2_ levels and changes in fire regimes can also alter grassland ecosystem function and negatively impact the intake and quality of grazing animals’ diets. Woody forages are harder for cattle and sheep to physically access as compared to goats, are less palatable, and have lower dry matter and protein digestibility compared to herbaceous plants (Archer et al., 2017).

The effects of increasing tropospheric O_3_ on plant productivity at scale and the range of potential secondary effects it might have (e.g. on weeds, pests and diseases, interactions with chemicals such as pesticides) have received less attention than eCO_2_ (Ainsworth et al., 2020). However, synthesis of crop responses to O_3_ finds that O_3_ pollution reduces crop yields to a similar level as nutrient, heat and aridity stress (Mills et al., 2018). For instance, using historical ground-level monitoring data, McGrath et al. (2015) estimated that, over the past 30 years, O_3_ pollution reduced U.S. soybean and maize yields by 5–10%. Ainsworth et al. (2020) provide a review of the literature on the effect of this air pollutant on plant productivity. The topic is not yet fully understood and remains one of the key uncertainties in crop, grassland and other global terrestrial models, with significant implications on our ability to predict future atmospheric composition and global climate, net primary productivity and provision of ecosystem services.

##### 2.1.1.2 Direct impacts of water and temperature on feed

Changes in temperature and water availability can greatly affect forage and crop yields and feed quality. Sensitivity to changes in climate depends on the crop type and other environmental factors, but there is strong agreement that air temperatures above approximately 30**°**C–34**°**C generally depress cereal yields under water-limited conditions, through accelerating crop development and damaging plant cells (Carlson, 1990; D. B. Lobell et al., 2011; Meerburg et al., 2009). The maximum temperature for growth of temperate legumes and pastures is around 30–35**°**C, increasing to 35–50**°**C for tropical species (Ludlow, 1980). High temperatures are often coupled with water stress, since low soil moisture results in a decrease in evaporative cooling from the landscape (Mueller and Seneviratne, 2012) and high temperatures increase crop water loss (Lobell et al., 2013). The combination of warmer temperatures and drier conditions tends to favour C_4_ rather than C_3_ species (Hatfield et al., 2011; Izaurralde et al., 2011). The concentration of ergot alkaloids, and other potentially toxic secondary compounds (e.g., hydrogen cyanide in cassava and forage sorghum) are also likely to increase in response to a hotter and drier climate (Bourguignon et al., 2015; Brown et al., 2016; Gleadow et al., 2016).

Increased instability of feed supply is particularly a concern in grazing systems where it represents a major challenge for herd size and grazing intensity management (Godde et al., 2020; Sayre et al., 2013; Sloat et al., 2018). Pastures with high year-to-year precipitation variability were found to currently support lower livestock stocking rates than less variable regions (Sloat et al., 2018). Studies focused on grassland vegetation have also found that changes in seasonal climate patterns can have either positive or negative impacts on above ground biomass, depending on the nature of the change and the agro-ecological context (Craine et al., 2012; Guan et al., 2014; Peng et al., 2013; Prevéy and Seastedt, 2014; von Wehrden et al., 2010; Zeppel et al., 2014). The arrangement of climate extreme sequences such as drought sequences or number of hot days in a row, could have significant implications for the livestock sector (Stafford Smith and Foran, 1992). While less commonly researched than droughts, other hazards such as fires, heavy storms, flooding events, surface melt and icing events, as well as the appearance of new lakes, streams and marshes also disturb crop growth, reduce arable land and restrict animal access to pastures (Amstislavski et al., 2013; Pan et al., 2019). For instance, in northern Russia, nomadic reindeer herders migrate hundreds of kilometres in spring and autumn to connect summer and winter pastures. The appearance of new water bodies and change in size of existing ones due to melting permafrost can act as barriers, changing migration routes and increasing grazing pressure on the most accessible pastures (Amstislavski et al., 2013).

Changes in precipitation patterns in saline areas will also affect soil salinity and agricultural production potentials. Salinity intrusion and associated reductions in forage area have led farmers across the coastal belt in Bangladesh to look for other sources of livestock feed (Alam et al., 2017). Tajul Baharuddin et al. (2013) suggest that the predicted local sea-level rise for areas such as Carey Island in Malaysia would prevent oil palm production by the 21st century, due to seawater intrusion. This has implications for livestock production through potential reductions in the production of palm kernel meal, which is often fed to cattle in industrial systems. Integrated palm-cattle systems, where cattle graze under trees or are fed palm fronds removed as part of plantation maintenance, would also be impacted. Increases in the frequency, duration and intensity of heavy rainfall events, drought periods and sea level rise will also increase exposure of water, croplands and grasslands to soil contaminants with potential harmful impacts for crop and forage yield quantity and quality (Biswas et al., 2018; Lemonte et al., 2017; Marrugo-Negrete et al., 2019).

##### 2.1.1.3 Feed yields, as projected in the future by biophysical models

At higher levels of warming, crop yields are projected to drop, especially at lower latitudes (Rosenzweig et al., 2014). This is particularly the case for maize and wheat yields which begin to decline with 1 °C–2 °C of local warming in the tropics, and drop by up to 60% under 5 °C of local warming (IPCC, 2014a). Temperate maize is less clearly affected at the 1–⁠2 °C threshold, but would be significantly affected with warming of 3 °C–5 °C. Recent studies also show that global food production has likely already been impacted (Asseng et al., 2015; Lobell et al., 2011; Ray et al., 2019). Ray et al. (2019) estimated that the impact of observed climate change on yields of different crops ranged from −13.4% (oil palm) to +3.5% (soybean), with impacts mostly negative in Europe, Southern Africa and Australia but generally positive in Latin America. Crop yield interannual variability is likely to progressively increase in many regions (IPCC, 2014a). For instance, Müller and Robertson (2014), in a gridded modelling study reported an increase of interannual variability of more than 5% in 64% of grid cells, and a decrease of more than 5% in 29% of cases by 2050.

Regarding forage availability, as for food crops, the diversity and severity of likely impacts differ considerably by location and species. In an assessment of global rangelands, Godde et al. (2020) found that global mean herbaceous biomass is projected to decrease of 4.7% by 2050 under RCP 8.5, with 74% of global rangeland area projected to experience a decline in mean biomass. The largest regional decrease was projected for Oceania while the highest increase was found for Europe. Another study focussed on European grasslands (Chang et al., 2017) also found projected increases in grassland productivity, mainly attributed to the simulated fertilisation effect of rising CO_2_. Both studies highlight projected increases in biomass inter-annual variability over some regions. Woody encroachment is also projected to occur on over 51% of global rangeland area by 2050 under RCP 8.5 according to Godde et al. (2020).

In an integrated partial equilibrium modelling approach, Havlík et al. (2015) found that the climate change impacts on crop and pasture forage yields will have little effect on global milk and meat production by 2050 due to trade in animal products, which could compensate for the feed deficits in some parts of the world. However, depending on the scenario, the impacts could be more pronounced at the regional scale. The most uncertain and potentially the most severe effects were found for sub-Saharan Africa, where for example, ruminant meat production could increase by 20% by 2050 but could also decrease by 17%, depending on projected feed supply based on varying the biophysical crop model and CO_2_ fertilisation assumptions.

Yield projections as above-mentioned are subject to large uncertainties. Models do not usually consider extreme events and overall increasing variability, or explicitly represent adaptation or effects such as changes in tropospheric O_3_, pests, pollinators, or agricultural labour. There are also large uncertainties as to climate extremes and trends in the future (Eyring et al., 2019; Sillmann et al., 2017) and changes in management practices, historical and projected land-use patterns (Polley et al., 2017). Our understanding of ecosystems responses to climate change is also limited (Rosenzweig et al., 2014; Schewe et al., 2019), including the interacting consequences of changes in temperature, precipitation, eCO_2_ and tropospheric O_3_, particularly in the context where management-driven yields increases are still occurring across vast areas of croplands. Possible ecosystems transitions from equilibrium to non-equilibrium systems driven primarily by stochastic abiotic factors will likely result in highly variable and less predictable primary production. Land uses such as grazing can also regulate grasslands responses to climate change. For example, sheep grazing has been found to limit CO_2_ stimulation of grassland productivity by selectively consuming legumes and forbs, plants with the greatest growth responses to CO_2_ (Newton et al., 2014). The issue of uncertainty is even more significant for grass yield projections than for crops, as reference data are less available for models’ development and evaluation.

##### 2.1.1.4 Impacts of pests, pathogens, weeds and pollinators on yields

The effect of climate hazards on pests (e.g. insect pests, pathogens), weed outbreaks and pollinators can have significant consequences for animal feed availability as reviewed in Myers et al. (2017).

Pests, pathogens and weeds are estimated to currently reduce the production of major crops by 25–40% (Flood, 2010). Increases in temperature increase winter survival of insect pests and rates of herbivory (Bale et al., 2002), and alter the spatial distribution of pests and pathogens. Bebber et al. (2013) reported an average poleward shift of pest and pathogen distribution of 2.7 km per year since 1960, though there is substantial variation among taxonomic groups. Under climate change, the spatial mismatches between pests and natural predators may be exacerbated in some regions, weakening biological control systems (Selvaraj et al., 2013). In some instances, weather extremes can weaken crop defences and create niches for pests and weed outbreaks (Rosenzweig et al., 2001). In other cases, extreme events can reduce pests and weeds, and as such support crop establishment and growth (Young, 2015). Recent intense desert locust outbreaks across East Africa, Asia and the Middle East have been linked to a series of cyclones causing warm and wet conditions (Salih et al., 2020). In Ethiopia, as per April 2020, nearly 200,000 ha of cropland were damaged by the insects, leading to the loss of over 356,000 tons of grain and thousands of tons of crop residues, a key livestock feed in the country (FAO, 2020). An additional 1.3 million hectares of pasture were affected, reducing pastoral areas by as much as 61% in the Somali region. Increases in eCO2 concentrations may also influence the weed composition and crop defences in complex ways (Zvereva and Kozlov, 2006), including reducing the effectiveness of herbicides (Ziska and Goins, 2006; Ziska and George, 2004). Shifting pest and disease patterns may increase the use of pesticides, some of which (i.e. dioxins) can pass on to animal products. These toxins can remain in soils for extended periods, and can contaminate animal feeds and water sources, particularly in conditions with alternating periods of drought and floods that are more likely with climate change (van der Spiegel et al., 2012).

More unstable weather, including more humid and cloudier conditions will lead to more on-farm post-harvest losses of animal feed, especially in developing countries with hot climates where most smallholders rely on the sun to dry their crops and forages before storage (Hodges et al., 2011). Contamination by toxins will likely also be higher (van der Spiegel et al., 2012). For instance, maize and sorghum can become contaminated by aflatoxins, particularly in drought conditions. While concentrations at harvest are usually not poisonous, the storage of grains under damp or poorly aerated conditions can lead to mould and the poisoning of animals and consumers. Increases in pest infestation frequency or intensity under climate change will also result in higher crop losses where storage facilities are inadequate.

While all grasses and most staple food grains such as maize, wheat, rice and sorghum are wind or self-pollinated, some crops used as livestock feed in industrial and mixed crop-livestock systems are animal pollinator-dependent to varying levels (e.g. soybean, cowpeas, pigeon peas, broad beans, rapeseed, oilseed rape, oil palm and some vegetable and fruit crops) (Klein et al., 2007). Climate change impacts on pollinators include changes in the abundance and distribution of both flowering plants and pollinators (Abrol, 2011; Hegland et al., 2009; IPBES, 2016; Memmott et al., 2007), and the timing of flowering and pollinator emergence and migration (Parmesan and Yohe, 2003), causing a mismatch in pollinator availability and crops to be pollinated. This contributes to reductions in the breadth and nutritional value of feed for pollinators (Ziska et al., 2016), which in turn decreases pollinator abundance. Increases in eCO2 concentrations also affect the nutritional value of key forages for pollinators (Ziska et al., 2016). While the net effect of climate change on pollinators remains uncertain, studies indicate that a reduction in animal pollination would decrease yields of numerous pollinator-dependent food crops (Klein et al., 2007).

#### 2.1.2 Water availability and quality

Water is used at various stages of the livestock supply chain: to grow feed; for animal consumption and cooling; to produce electricity, fertilizers, pesticides and fuel; and to clean animals and infrastructure (see detailed inventory in FAO (2019b)). Most water in the livestock value chain is used for feed production, accounting for over 90% of water consumption in many systems (Legesse et al., 2017; Mekonnen and Hoekstra, 2012). The amount of water required for livestock consumption varies with local climate conditions, with higher consumption under hot conditions (Ward and McKague, 2019). For instance, once air temperatures exceed 30 °C, the expected poultry drinking water intake can increase by 50% above normal rates. In arid and semi-arid regions, the increased frequency, intensity and duration of droughts are a significant challenge for sustaining water supply to livestock and feed production.

Climate change is also projected to reduce raw water quality (Jiménez Cisneros et al., 2014), which can decrease animal water intake, feed intake and health (The State of Victoria, 2018; Valente-Campos et al., 2019). Poor local water quality can be caused by warmer temperatures, sea level rise, or higher sediment, nutrient and pollutant loadings due to heavy rainfall. Reduced dilution of pollutants during droughts and disruptions of treatment facilities during floods are also a concern. High salt concentrations in water and feed depress livestock feed intake and production (Masters et al., 2006; Sharma et al., 2016). The tolerance of animals to different levels of total dissolved solids (including inorganic salts) in water varies considerably among varied species with poultry, buffaloes and dairy cattle having lower tolerance levels (desirable maximum concentration for healthy growth of 2000–2500 ppm) than beef cattle, sheep and pigs (4000 ppm, Sharma et al., 2016).

#### 2.1.3 Shifts in agricultural lands

The effects of climate change on resources, as mentioned in this section, will continue to lead to shifts in the global agricultural area as well as changes in seasonality and crop and livestock suitability. For example, King et al. (2018) estimate a northward shift of the feasible agriculture zone by up to 1200 km by the end of the century, although many of these areas are associated with highly variable water balances. The world's drylands have expanded over the last 60 years, mostly in semiarid regions, and are projected to expand further during this century (Huang et al., 2017). In sub-Saharan Africa, more than 20% of the mixed crop-livestock system in arid and semi-arid regions is projected to become unviable for crop agriculture by mid-century (Jones and Thornton, 2009). Grass yields may overall be less impacted by climate change than crop yields, which may favour grazing systems and potentially change the current trend towards more intensive systems (Havlík et al., 2015).

### 2.2 Animal health and production

The impacts of climate change on animal growth, production and welfare include impacts mediated by reduced feed intake, direct physiological and metabolic effects, and changes in behaviour (e.g. Filipe et al. (2020), Gauly and Ammer (2020), Lara and Rostagno (2013), Lees et al. (2019), Nardone et al. (2010), Polsky and von Keyserlingk (2017), Romo-Barron et al. (2019), Saeed et al. (2019), Sevi and Caroprese (2012), Tedeschi and Fox (2020)). The potential impacts of climate change on livestock health and production are already well-reviewed in the literature, and we highlight key effects below.

#### 2.2.1 Heat stress

Heat stress is primarily caused by exposure to high ambient temperatures and relative humidity, which limit the capacity of livestock to shed heat to their environment, on farm or during transportation (see section 2.3.1). Vulnerability to heat stress varies according to species, breed, life stage, genetic potential, nutritional status, size, level of insulation (hide thickness or distribution of feathers) and previous exposure of the animal, with animals with high energy demands (i.e. high-yielding individuals and breeds) most susceptible (Bernabucci et al., 2010; Rashamol et al., 2019; Saeed et al., 2019). For example, dairy cows are more susceptible than beef cattle, and temperate *Bos taurus* breeds are more susceptible than tropically adapted *Bos indicus* cattle and their crosses (Polsky and von Keyserlingk, 2017).

In most cases, the impact of heat stress is reduced productivity and animal welfare. However, under severe or prolonged conditions, mortalities will also occur. Reduced feed intake is one of the first and biggest consequences of heat stress, leading to declines in growth rates and production of milk or eggs.

Heat stress also affects livestock production through changes to fertility and susceptibility to disease. In both mammals and poultry, the impacts on fertility are caused by reduced ovarian function, reduced motility of spermatozoa, and inhibition of embryonic development (Nawab et al., 2018; Polsky and von Keyserlingk, 2017; St-Pierre et al., 2003). Cattle also show reduced expression of estrus behaviour, further reducing the chances of reproductive success. In addition, heat stress may depress immune function and the effectiveness of some vaccines (Bagath et al., 2019; Hirakawa et al., 2020), increasing the incidence of livestock disease. The reverse is also true, with animals showing clinical signs of disease more susceptible to heat stress (Gaughan et al., 2008). While covered to a limited extent in the literature, there is some evidence that high temperatures may decrease the ability of mammalian herbivores to detoxify plant secondary compounds (Dearing, 2013; Moore et al., 2015). While most work to date has been done with rodents and wildlife (e.g. Kurnath et al. (2016)), high environmental temperatures have been shown to increase the susceptibility of cattle to poisoning from ergot alkaloids in tall fescue (Aldrich et al., 1993).

The impacts of heat stress on livestock can be both immediate and long-lasting, and can also affect offspring exposed to heat stress *in utero*. Research in dairy cattle and pigs has shown that heat stress *in utero* reduces milk yield at first lactation (Dahl et al., 2016; Monteiro et al., 2016), and alters nutrient partitioning and carcass composition (Boddicker et al., 2014; Johnson et al., 2015). Animals exposed to heat stress *in utero* may also be better adapted to heat stress conditions at maturity, with Ahmed et al. (2017) reporting that cows exposed to heat stress *in utero* are better able to regulate core body temperature.

Heat stress can also impact the quality of animal products, reducing the size of eggs and thickness of eggshells (Mashaly et al., 2004), decreasing the fat and protein content of milk (Bernabucci et al., 2002; Sevi and Caroprese, 2012), and changing the colour and water-holding capacity of both red and white meat (Gonzalez-Rivas et al., 2020). These changes can make livestock products less appealing to customers, increase wastage, and reduce the price that producers receive for their products. Selection of breeds that are better adapted to high temperatures can also have implications for product quality; while *Bos indicus* cattle are better adapted to high temperatures and humidity than *Bos taurus*, their meat is less tender (Crouse et al., 1989; Johnson et al., 1990), and tends to score lower in meat quality assurance schemes such as the Meat Standards Australia index, receiving lower prices in some markets.

Heat stress, and other stressors such as feed withdrawal can also cause the dissemination of enteric pathogens such as Salmonella, *Escherichia coli* O157:H7, and *Campylobacter* from livestock into human food, and as such is also a major health concern. Indeed, these external stressors can increase animal pathogen carriage and shedding as reviewed in Gonzalez-Rivas et al. (2020). The underlying mechanisms have however not yet been fully explained.

#### 2.2.2 Other impacts

In addition to heat stress, the increased frequency and intensity of storms, fires, floods and cold waves can have significant repercussions for the livestock sector. Extreme flooding followed by cold weather in northern Australia in early 2019 caused the loss of approximately half a million livestock (mostly cattle, but also sheep, goats and horses) (Queensland Government, 2019). Similarly, in October 2018, Hurricane Michael destroyed an estimated 84 chicken houses and killed over 2 million chickens in Georgia, U.S (Georgia Department of Agriculture, 2018). In geographic areas with cold winters, such as in the Northeast US, warmer temperatures may also reduce animal cold stress and maintenance energy requirements, as well as housing heating (Hristov et al., 2018; Toghiani et al., 2020).

Climate change may also impact infectious livestock diseases by changing their spatial distributions, affecting annual and seasonal cycles, altering disease incidence and severity, and modifying susceptibility of livestock to illness (Bagath et al., 2019; Filipe et al., 2020; McIntyre et al., 2017; Patterson and Guerin, 2013; White et al., 2003). Many infectious pathogens that cause disease in livestock are sensitive to changes in climate, primarily moisture, rainfall, temperature and particulate matter – many of these diseases are zoonotic (McIntyre et al., 2017). Some pathogen transmission routes are also more climate-sensitive than others. Vector-borne, foodborne, water-borne and soil-borne pathogens are the most likely to be affected by climate change, while those transmitted directly or by fomite are the least likely to be affected by climate change (McIntyre et al., 2017). In particular, the quantity and spread of insect vectors such as flies, ticks and mosquitoes, but also wild birds, rodents and mammals which can transmit disease to farmed poultry, pigs and ruminants is of concern to the livestock sector.

It is also probable that increases in tropospheric O_3_ will affect animal health (Menzel, 1984), though increases in mortality are likely smaller than those reported for heat stress events (Egberts et al., 2019).

### 2.3 Processing operations, storage, transport, retailing

#### 2.3.1 Live animal transportation

Livestock are often transported long distances by road or sea to markets and slaughter. Climate change, particularly increases in temperature and climate-driven disruptions in the transport network, may increase the risk of heat stress during transport, which in turn, can contribute to poor animal welfare and deaths (Caulfield et al., 2014; Collins et al., 2018). This aspect is not often considered in heat stress literature reviews. High stocking density of animals during transport increases ambient temperature and humidity, and reduces ventilation, decreasing opportunities to shed heat loads. In addition, animals transported by truck usually do not have access to water on-board and can become dehydrated faster during hot conditions. In response to this, the live export of sheep from Western Australia to the northern hemisphere is currently prohibited in summer (June to September) (Australian Department of Agriculture and Water Resources, 2019). Increased scrutiny of animal welfare in other regions may result in additional restrictions on animal transport such as when animals can be moved (time of day and year), and the duration of transport.

#### 2.3.2 Built-up capital

Higher temperatures, increased humidity, increased frequency of extreme weather events, and rising sea levels put additional stress on built-up capital (machinery, transportation infrastructure, electricity networks, telecommunications, etc.). This will lower productivity across many economic sectors, increase construction, operating and maintenance costs, and may shorten the lifespan of critical industry infrastructures (Lloyds, 2015; Schweikert et al, 2014a, 2014b; Wang et al., 2012).

Damage and degradation of key transportation infrastructure such as roads, railways, and port infrastructure are of particular concern for the livestock sector (Markolf et al., 2019). For example, in Australia, 95% of livestock are transported domestically by road from farms to central points such as saleyards, feedlots, abattoirs or ports of embarkation for live export (Fisher et al., 2006). In early 2019, cattle producers in northern Australia reported over 29,000 km of farm roads destroyed by extreme flooding, in addition to the loss of tools, machinery, homes and 22,000 km of fencing (Queensland Government, 2019). At a sea level rise of 1.1 m (high end scenario for 2100), the Australian Government projects that coastal assets at risk from the combined impact of inundation and shoreline recession are greater than AU$226 billion and include between 27,000 and 35,000 km of roads and rail, with a value of between AU$51 and AU$67 billion (Commonwealth of Australia, 2011). In the EU, almost 90% of external trade is transported by sea (Suárez-Alemán, 2016) and studies found that 64% of all European seaports are expected to be affected by inundation events by 2060, caused by global mean sea level increases and combined effects of tides, local waves, and storm surges (Christodouloum and Demirel, 2017). Reduced polar ice may offer new trade routes that could reduce travel time between Europe and the Northern Pacific (Lindstad et al., 2016), and could reduce the reliance on several key trade passages (e.g. Panama Canal, Suez Canal, and Strait of Malacca) but could also threaten new marine ecosystems through the introduction of invasive species (Miller and Ruiz, 2014).

#### 2.3.3 High temperatures, product distribution and storage

Increasing temperature results in worsening conditions for storage, which leads to degradation of food quality and shelf-life, increased wastage and increases the likelihood of the proliferation of microbes and fungi (i.e. *E. coli*, salmonella, aflatoxin and mycotoxin producing organisms, etc.), especially under humid conditions (van der Spiegel et al., 2012). Even in developed economies, increased temperatures have been found to increase the likelihood of food poisoning, suggesting that increasing temperatures will lead to reduced food quality and safety (D'Souza et al., 2004). For instance, chilled (below 10 °C) storage life is halved for each 2–3 °C rise in temperature (James and James, 2010). In addition, where climate change results in higher levels of microorganisms on meats and produce prior to processing, storage temperatures may need to be lower to preserve required shelf-lives.

Climate control (air conditioning and refrigeration) can mitigate some of the negative impacts of climate change on food processing and distribution. However, this leads to increased energy usage and costs, and current levels of refrigeration are already estimated to contribute 1% of global CO_2_ emissions (James and James, 2010). Sarhadian (2004) found that an increased ambient temperature from 17 to 25 °C resulted in an 11% increase in average power consumed in a catering establishment. As a food moves along the cold chain it becomes increasingly difficult to control and maintain its temperature. This is because the temperatures of bulk packs of refrigerated product in large storerooms are far less sensitive to small heat inputs than single consumer packs in open display cases or in a domestic refrigerator or freezer (James and James, 2010). Fresh milk, in particular, requires significant amounts of cooling and could be the most impacted by increasing costs, or deterioration of cold chains.

Globally, the use of refrigeration is also far below what is optimal, with only 10–20% of perishable value chains refrigerated (Coulomb, 2008). Low- and middle-income (LMIC) countries in already hot environments are particularly vulnerable, due to limited cold chain development. These are also the regions that have seen the largest increases in consumption of livestock products in recent years (FAOSTAT, 2020; Herrero et al., 2018). It is estimated that if LMIC acquired the same level of refrigerated equipment as that in high income countries, wastage of perishable food would decrease by over 200 million tonnes, or 14% of the current consumption in these countries (International Institute of Refrigeration, 2009).

#### 2.3.4 Extreme events, products distribution and storage

Changes to the frequency, intensity and duration of extreme events will impact supply chains by increasing uncertainty in input flow and damaging infrastructure. This will make trade patterns less regular, increasing reliance on complex logistic systems. Extended droughts reduce hydropower generation, and can lead to increased wildfires, both of which can lead to brownouts, a particular threat to supply chains heavily reliant on temperature controls to maintain food quality and safety. Extreme events can also damage key transportation (road, rail, ports) infrastructure, limiting the distribution of products. Both low and high flow can affect the navigability of key inland waterways like the Mississippi River (Olsen et al., 2005) and Great Lakes in North America (Attavanich et al., 2013). For instance, a major drought in 1988 drastically reduced barge movement along the Mississippi a critical corridor for transporting grains in the USA to the port of New Orleans (Changnon, 1989).

In many countries the supply chain for livestock products is highly concentrated and coordinated, and disruptions anywhere along the supply chain can have impacts throughout the chain. For example, the COVID-19 outbreak in the USA prevented the processing of pigs, which forced pork farmers to either hold pigs, or cull them to avoid paying to feed them (BBC, 2020). There is more scope for keeping animal stocks for beef, but any culling in herds also has a longer impact on animal stocks due to lower reproductive capacity of cattle compared with chickens or pigs.

### 2.4 Livestock product consumption

We highlight in previous sections the potential impacts of climate change on the supply and processing of animal products, as well as on the quality and safety of animal products through contamination with pathogens or pesticide (sections 2.1.1.4, 2.3.3) and reduced nutritional quality and sensory appeal (section 2.2.1).

The social licence of the livestock sector is increasingly challenged by the rising awareness of climate change and livestock impacts. These societal changes are also influenced by animal welfare and environmental concerns, and increasingly supported by civil society, governments and other institutions. Changing social norms are likely to impact diets, especially in high-income countries (Godfray et al., 2018). Research also found that temperature changes can affect dietary preferences of consumers (Motoki et al., 2018; Needs et al., 1993), although the effects on the consumption of livestock products remain largely understudied. Changes in the price of animal products and relative magnitude of price changes across commodities will also alter consumption patterns (Muhammad et al., 2017; Valin et al., 2014). These changes will be driven by product availability and quality, as well as costs along the supply chain. The rate of growth in gross domestic product, which can be influenced by climate change (Burke et al., 2015), will also alter consumers' purchasing patterns. Indeed, food price sensitivity tends to fall as incomes rise, and a smaller share of income is spent on food (i.e. Engel's Law; Clements and Si (2018)). Sensitivity to primary commodity prices may also decline as supply chains complexify: increasingly the cost of the food is driven by value-added processing, packaging, and branding. For example, the farm share of the food value added in the European Union has fallen from 31% in 1995 to 24% in 2005 (European Commission, 2009).

### 2.5 Labour

Labour availability and productivity are key determinants of the efficiency of the livestock supply chain. This is especially the case in less developed regions that do not rely on mechanization. However, human performance and health can be limited by a broad range of direct and indirect effects of climate change as reviewed in previous studies (Patz et al., 2005; Smith et al., 2014). These impacts relate to temperature, floods and storms, ultraviolet radiation, infections, air quality, nutrition, occupational health, mental health, violence and conflicts. For instance, in 2010, inhalation of climate-altering pollutants other than CO_2_ caused more than 7% of the global burden of disease (Smith et al., 2014). We further detail below two key drivers of labour productivity of particular concern to agricultural labour: heat stress and diseases.

Above 24–26 °C, labour productivity declines (International Labour Organization, 2019). At 33–34 °C, a worker operating at moderate work intensity loses 50% of their work capacity. In addition, exposure to excessive heat levels can lead to heatstroke and sometimes fatal outcomes. Productivity may decrease by 11–27% by 2080 in hot regions such as Asia and the Caribbean (Kjellstrom et al., 2009), and labour productivity for high intensity work declines by up to 31%–⁠38% (RCP 4.5–⁠RCP 8.5) in Southeast Asia and the Middle East by 2050, relative to a 2050 baseline without climate change (Knittel et al., 2020). In some areas, 30–⁠40% of annual daylight hours will become too hot for work to be carried out (Kjellstrom et al., 2016). Literature focussed on agricultural labour emphasises the challenges caused by physical labour under high temperatures, with dehydration often being the main cause of lost labour productivity (Wagoner et al., 2020; Wästerlund, 2018). Heat stress is of particular concern in production systems that are dependent on high inputs of human labour and located in environments that are already hot, such as smallholders in sub-Saharan Africa, as highlighted in Frimpong et al. (2017) and Yengoh and Ardö (2020). Foreign workers, who represent a large part of the agricultural labour force in some countries such as in the U.S. are also particularly at risk. Foreign farmworkers may have entered the country illegally, often do not speak the local language, have low education levels and incomes and limited access to health care. They often feel they have little control of their workplace, and tend to be reluctant to complain about unsafe work environments (Lambar and Thomas, 2019).

In addition to heat stress, communicable diseases such as Malaria, dengue fever, tick borne encephalitis, borreliosis, salmonellosis, cholera and bluetongue are all affected by climate change and affect the health of people working along the livestock supply chain (Caminade et al., 2019; Wu et al., 2016; World Health Organization, 2014). For instance, excessive rainfall and high temperature results in an increased transmission rate, reproduction rate and the proliferation of the *Plasmodium* species that causes malaria (Braide et al., 2020), and outbreaks of leptospirosis in Sub Saharan Africa (Lau et al., 2010). In areas where there is inadequate access to water and sanitation, flooding increases people's exposure to salmonellosis and cholera. Conversely, a drying climate and extended periods of drought in Australia have been conducive to the spread of Q-fever, with the disease-causing bacteria *Coxiella burnetii* able to survive and travel long distances on dust particles contaminated with animal faeces or birth fluids (Archibald, 2019). Warming temperatures in northern latitudes have also been reported to favour the distribution of anthrax, particularly in association with increased livestock densities (Walsh et al., 2018). Populations in close contact with livestock and wildlife are particularly at risk of zoonotic pathogens, with climate change further contributing to the risk of zoonosis (McIntyre et al., 2017).

### 2.6 Prices

Climate-related challenges from resource availability to consumption levels mentioned above can result in rises in costs of water, feeding, housing, storing, transport, retailing and insurance, which negatively impact actors throughout the livestock supply chain. An ensemble of crop and economic models with various representations of the global food system and associated assumptions (e.g. related to technological change, land-use policies or consumption patterns) projects a 1–29% increase in cereal prices by 2050 across Shared Socio-economic Pathways 1, 2, and 3 under climate change (RCP 6) (Hasegawa et al., 2018). This would impact consumers globally through higher food prices, with effects varying regionally. The price of animal products is also projected to increase, but projected prices are about half that of cereals, highlighting that in these models, the impacts of climate change on animal products are felt mainly through changes in costs and availability of feed. These results also highlight scope for feed substitution within the livestock sector. Most climate studies have focused on price levels and not on price volatility. However, with increased spatial and temporal variability in production and supply chain efficiency, it is probable that food prices will be more volatile under climate change (Mbow et al., 2019). The international agricultural trade system is dominated by regional trade (i.e. within Europe and Mediterranean Basin, North America, etc.), with trans-regional trade dominated by a few major international exporters (e.g. soybeans from Brazil, maize from the US, rice from Southeast Asia, lamb from New Zealand-lamb, etc., Herrero et al., 2018). Major shocks to these major producers could dramatically increase the volatility of international markets and reduce the reliable supply not only of food but also of feeds, which are traded at much higher volumes than livestock products. Furthermore, it can drive policy responses to protect domestic supply that can exacerbate food crises (Headey and Fan, 2010).

### 2.7 Social dynamics and systems change

Climate change is contributing to a range of societal changes with implications for the livestock sector. As noted above, changes in climate conditions will challenge productivity, economic viability, structure, and attractiveness of livestock-related enterprises with consequences on the availability, quality, affordability, safety and sensory appeal of livestock products. Climate change will likely amplify existing inequalities with particularly negative impacts on small-scale enterprises, which may struggle to remain competitive (Hallegatte and Rozenberg, 2017; Islam and Winkel, 2017). Social learning, the process of learning from and of responding to experience, will also shape social-ecological systems (Morrison et al., 2017). The mitigation and adaptation actions that food system actors themselves take may also affect social dynamics. For example, rising awareness of the negative consequences of climate change and the sector's environmental footprint may contribute to shifting social norms, particularly in high-income countries, that may favour less resource intensive production (Godfray et al., 2018), and encourage the further development of novel protein products (plant-based meats, cultured meats, etc.). All these changes will impact both local and global food systems dynamics and food environments.

## 3 Vulnerability of livestock-based socio-economic systems

The ability of the livestock sector to cope with or adapt to climate change, i.e., its adaptive capacity, depends on a range of socio-economic, political, institutional and environmental factors from local to global scales that interact in complex ways. Well known demographic, social and economic factors are driving change in the livestock sector. These factors are briefly presented below and treated in more depth in the Supplementary Information.

A growing human population, together with increasing incomes and shifts in dietary preferences, predominantly in low- and middle-income countries, are driving continued growth in demand for livestock products (especially poultry and pork) and resources needed to produce them, including land and water (FAOSTAT, 2020; Herrero et al., 2018). Potential enforced production systems transitions due to changes in land suitability will affect the food production landscape (Thornton et al., 2019). Large-scale demand for plant-based diets and production of synthetic livestock products may also alter livestock production systems dynamics (Springmann et al., 2018). The development of such food alternatives will be influenced by changing social norms and opportunity-costs of different food production systems under climate change and other global trends. In addition, competition between crop as livestock feed and crop for direct human consumption or biofuel production are increasing (Muscat et al., 2020). Human migration, partly induced by climate change, can also increase local pressures on scarce natural resources (FAO, 2018b). Rising food safety standards and governments' investments in infrastructure, price support schemes, taxes, credits, subsidies, input and output quotas and the health system, will also influence the vulnerability of livestock supply chains. For instance, the lack of infrastructure and access to knowledge, land, financial services, markets, new dynamic sectors and policy dialogues in rural areas is making the agricultural sector unattractive to youth, with negative implications for the agricultural sector's performance and adaptive capacity (FAO, 2014; Proctor and Lucchesi, 2012; White, 2012). Production challenges associated with harsh climates can also increase youth reluctance to enter or remain in agriculture. Discrimination based on gender, ethnicity, caste, and wealth impedes participation in markets, legal recognition of land and asset ownership, and other rights that play key roles in the ability of stakeholders to adapt to climate change. Civil conflict and institutional disregard of traditional knowledge, institutions and customary practices can also weaken the resilience of livestock systems (FAO, 2018b; IPCC, 2014b; Nielsen et al., 2020). While some of the political and institutional forces may contribute to persistent trends (e.g. continued exclusion of pastoralists from political processes), others may result in dramatic shifts in the near future and greatly alter the ‘vulnerability landscape’. The pace at which governance factors can change has been exemplified by governments' actions to stop the spread of diseases (e.g. H5N1 or COVID-19).

People in different production systems have differentiated exposures and vulnerabilities. Table 2, Table 3 highlight the exposure of people in different regions and production systems based on economic and livelihood indicators of the livestock sector. We summarise in Table 4 key vulnerabilities and adaptative capacity characteristics of people in grazing-only systems, mixed crop-livestock systems and industrial systems, as defined by Seré and Steinfeld (1996) and mapped in Robinson et al. (2011). These vulnerabilities are also discussed in Rivera-Ferre et al. (2016), and a focus on adaptation in mixed systems is provided in Thornton and Herrero (2015, 2014).

**Table 2:**
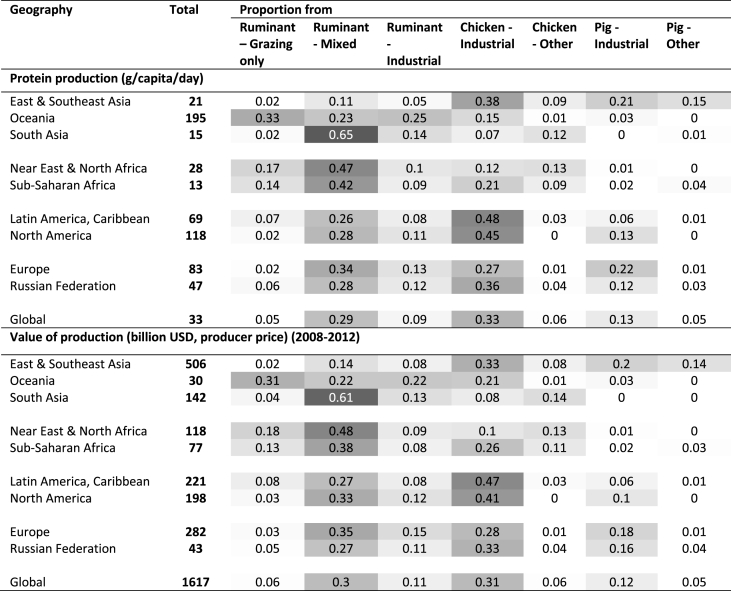
Land-based livestock economic and livelihood indicators – Total protein production and value of production per region and globally, and (shaded) relative contribution from different livestock production systems. The darker the shade, the higher the contribution. Livestock production systems as defined by Robinson et al. (2011). Calculations presented in the Supplementary information.

**Table 3:**
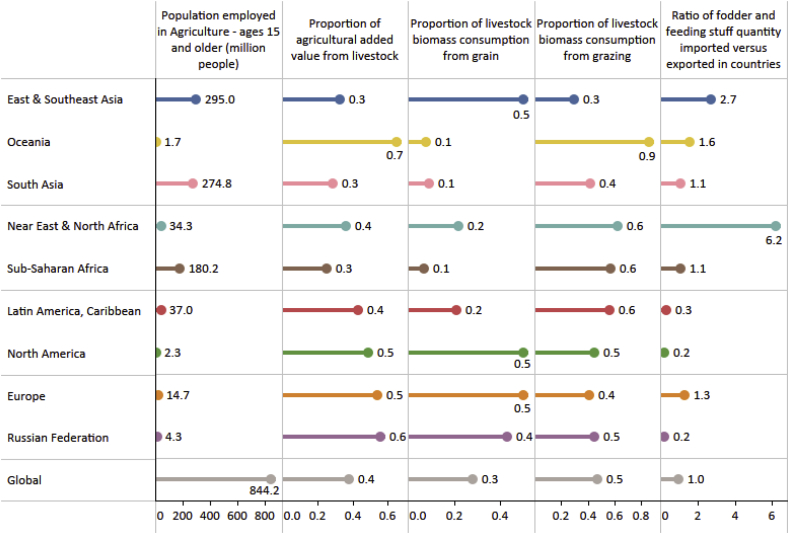
Land-based livestock economic and livelihood indicators at regional level. Calculations presented in the Supplementary Information.

**Table 4:** Some current key vulnerabilities and strengths of people in different livestock production systems. Livestock production systems as defined by Seré and Steinfeld (1996) and mapped in Robinson et al. (2011): livestock grazing systems (LG) and mixed systems (M) in arid (A), humid (H) and temperate (T) regions. Table adapted from Rivera-Ferre et al. (2016).

	Grazing systems	Mixed crop-livestock system	Industrial system
Vulnerability	• Political marginalization • Land encroachment • Land degradation • Land fragmentation • Remoteness • Reliance on physical labour related to limited mechanization • Lack of financial capital and alternative economic options • Conflicts (civil conflicts, conflicts over resources) • Land ownership and tenure arrangements (e.g. communal land tenure can limit land and infrastructure improvements)	• Limited mobility • Land degradation • Land scarcity especially from urban expansion • Rising food safety standards • Population growth • Economic margins often small and financial capital often low, resulting in lock-in • Economic competition favouring cropping • Co-managing price and climate variability • Learning and capital demands from having multiple farm components • Labour supply for peak periods of activity • Shrinking farm sizes	• Dependence on external inputs and hired labour • Energy intensive • Difficulties in re-locating built-up capital • Narrow gene pools in livestock and input crops • Large, high-yielding animals are more susceptible to heat stress and disease • High-yielding crops are often more sensitive to heat and water stress • Challenges in waste disposal and animal welfare impacting on social licence to operate • Susceptibility to disease outbreaks • Low economic margins • Operating close to or at maximum physiological and financial limits • Integrated in highly efficient value chains
Adaptation capacity	• Mobility to adapt to spatial and temporal climate variability • Family labour • Communal land and social collaboration • Local knowledge of diverse resources • Capacity to add value to marginal land via provision of ecosystem services • Wide livestock gene pool • Recycling plant nutrients • Transformation to mixed systems • Off farm income	• Integration of agriculture and livestock • Capacity to use crop residues • Often private land, hence have agency • Flexibility in crop-livestock allocation and other decisions • Diversification • Family labour • Wide livestock and forage gene pool • Recycling plant nutrients • Flexibility in allocating produce to subsistence or market • Off farm income	• Access to global feed and input supply chains • Access to credit and modern technology • Access to global consumer market • Capital mobility and exploiting economies of scale • Control of many aspects of the system • Good information systems (climate, financial, supply) allowing rapid responses

Some characteristics of grazing systems compared with other food production systems are their usual remoteness from population centers and limited access to information, markets, capital, labour and veterinary services. These characteristics, together with market price fluctuations and social considerations (e.g. benefit perception), limit opportunities for management options that could mitigate the effects of climate change and high climate variability such as timely stock adjustments. People in these systems, often both geographically and politically distant from policy makers, tend to be marginalised and receive limited investments from governments and businesses (Godber and Wall, 2014; Marshall, 2015; Nielsen et al., 2020; Sayre et al., 2013; Thomas and Twyman, 2005). Grazing systems are often located on land that is poorly suited for cropping, notably due to high climate variability. Herd mobility and collective resource management have been key strategies to take advantage of the spatial and temporal variation in water and forage availability. However, changes in land tenure and increasing landscape fragmentation have become a major concern for grazing systems resilience in many parts of the world (Hobbs et al., 2008; Reid et al., 2014). For instance, in Inner Mongolia, the culture had adapted to the harsh climate by using mobility, cooperation, and reciprocity strategies as described in Dalintai et al. (2012). However, collectivization between the 1950s and mid-1980s followed by market reforms in the early 1980s disrupted these traditions and weakened herd mobility. The state-driven nomad sedentarisation projects in China (Hruska et al., 2017) and transitions from communal to semi-commercial land tenures in southern African rangelands (Dube and Pickup, 2001) have also limited adaptive capacity of people in these regions. In Australia, where most grazing land is owned or leased, it is common practice to move cattle from a droughted area to other privately run properties that have adequate pasture, and graze for a fee (“agistment” strategy; McAllister, 2012; McAllister et al., 2006). The shortage of productive land during widespread droughts can however greatly limit the possibility and economic viability of such a mobility strategy.

Farmers in mixed crop-livestock systems often have more limited economic opportunities as compared to cropping systems (Rivera-Ferre et al., 2016). This is particularly the case for small-scale systems. Shrinking farm sizes limit opportunities for management adaptations and more stringent food safety standards limit the ability of smallholder farmers to enter expanding markets as these standards create new knowledge requirements, additional investments and stronger linkages between producers and buyers (Humphrey, 2017). The literature has extensively covered the impacts of climate change on crop enterprises in mixed systems, and to a lesser extent, the impacts on livestock enterprises (Thornton and Herrero, 2015), although the focus has often been on yields rather than livelihood outcomes or other sustainability dimensions (Ricciardi et al., 2020). However, relatively little is known about how crop-livestock interactions may be affected or offer buffering capacity to help smallholders adapt to climate change (Thornton and Herrero, 2015).

Industrial systems are generally based on high-yielding animals, which are more susceptible to heat stress, so require greater investment in infrastructure to insulate them from climate extremes. Compared to more extensive systems, the close physical proximity of animals to each other and humans can increase the possibility of disease outbreaks. These outbreaks can however be more quickly identified and contained. The limited contact with wildlife also reduces the risk of diseases from endemic carriers. Industrial systems tend to be energy and capital intensive which can make them vulnerable to disruptions to energy supply. Due to greater integration in efficient value chains, they are also more vulnerable to disruptions to transportation and demand given more limited capacity for long run inventory.

Overall, livestock ownership itself can support farmers resilience in times of climate change and climate extremes. This is particularly the case in smallholder farming systems, where livestock plays multiple roles beyond producing food for the market (Alary et al., 2015; CIRAD, 2016). These roles include provision of wool, hides, skins, manure, animal traction, transportation and food for home consumption. Livestock can also contribute to the diversification of income and accumulation of capital savings, and as such can also be used as a risk reduction strategy by vulnerable farming communities. In particular, livestock production is often more resilient to high climate variability or short length of growing periods than crop production (Robinson et al., 2011; Sloat et al., 2018). Animals can also be sold in times of climate shock to help compensate for crop failures, loss of income or additional expenses (WFP, 2019). Diversified livestock systems, including the ownership of multiple animal species and breeds, improve dietary adequacy and diversity (Jones, 2015; Murendo et al., 2018; Ritzema et al., 2019; Romeo et al., 2016) and food security (Bahadur KC et al., 2016). Shifts in animals breeds can serve as a climate adaptation strategy (e.g. shifts from cattle to camels in East and North Africa or from British *Bos Taurus* to Zebu *Bos indicus* in Australia, Bortolussi et al., 2005; Faye et al., 2012; Kagunyu and Wanjohi, 2014; Watson et al., 2016). Nevertheless, climate change may challenge the viability of livestock systems in some contexts, given environmental, socio-economic and institutional constraints to adaptation.

## 4 Societal risk of impacts on livestock supply chains - at the intersection of hazards, exposure and vulnerability

The risks of climate-related impacts arise from the interaction of climate-related hazards (including hazardous events and trends) with exposure of the livestock supply chain to these hazards and the vulnerability of the socio-economic systems within which they are embedded (Fig. 1).

Worldwide, rangeland communities that are projected to see the most negative impacts of climate change on vegetation are also amongst the communities most vulnerable, according to a range of socio-economic vulnerability indicators at local and national levels (see Supplementary Information, Godde et al. (2020)). Across a range of dimensions, including food security, health, socioeconomics, and governance, Godber and Wall (2014) found that the region with livestock-based food production at most risk was South Asia. They found that LMICs were generally more at risk than higher income regions, with the top decile of nations at risk located in Sub-Saharan Africa, seven of which were in Eastern Africa. The potential impacts emerging from such risks will affect all dimensions of sustainable livelihoods⁠—human, social, natural, physical, and financial capitals (Fig. 2).

Climate-related risks will be context specific, but also greatly influenced by global socio-economic trends and shocks. Differences in risks arise from climatic and non-climatic factors and from multidimensional inequalities, often due to uneven development processes. These context specificities highlight the importance of renewed attention to diversity within the livestock sector and its multiple socio-economic and environmental contributions. For instance, in Oceania, livestock represents 70% of agricultural added-value (Table 3) and over 90% of livestock feed is from grazing (Table 2, Table 3). Most of Oceania's grazing lands (mainly in Australia) are subject to high and increasing climate variability and frequent and intense fires, droughts and floods (Nielsen et al., 2020). As such, the viability of the sector will greatly depend on the success of the strategies implemented to cope with high climate variability. These strategies will need to account for geographic remoteness, limited economic margins, and over-grazing. Increasing climate variability and drying trends on grasslands is also a key concern in arid and semi-arid African regions (where 60% of feed is from grazing), as are changes in the length and start of the crop growing seasons (over 70% of protein production comes from ruminant mixed systems and grain-fed chicken; Table 2, Table 3; Thornton et al., 2011). Socio-economic and institutional support, including social safety nets, is urgently needed considering the multiple roles livestock plays in such regions. Overall, small-scale farmers in environments that are already hot and with limited resources for adaptation will be the most at risk. We estimate that 228.6 million people worldwide live in low-income countries in grazing-only and mixed crop-livestock regions in arid environments. Agriculture represents approximatively 57% of total employment in these countries (see Supplementary Information for calculations). Although the share is likely to decline in the future, in absolute terms, the number of vulnerable people employed in agriculture will remain high.

The impossibility of predicting complex interconnected biophysical and social systems far into the future challenges the evaluation and prioritisation of climate risk mitigation and management strategies. Given this fundamental uncertainty, it is crucial to consider a wide range of plausible futures and climate outcomes, which while it may not allow us to predict the future, can allow us to assess the range of possible impacts and to test the robustness of policies and interventions across alternative contexts (IPCC, 2014c).

## 5 Conclusion

The rapid growth of the livestock sector and its various contributions to the economy and human livelihoods highlight the importance of better understanding the impacts of climate change on livestock.

While it is certain that climate change will impact the sector throughout the food supply chain⁠—from farm production to processing operations, storage, transport, retailing and human consumption⁠—large uncertainties remain as to the nature, extent and magnitude of these impacts. These uncertainties relate to the future climate as well as exposures and responses of the interlinked human and natural systems to climatic changes over time. For instance, the impact of climate change on agricultural labour, on the livestock supply chain from farmgate to retailers or on livestock products prices and consumption behaviours have received limited attention in the past. The impacts of climate change on livestock feed production and quality, especially forages, have also been understudied as compared to key staple grains. In addition, the rates of change in global socio-economic and environmental drivers are such that the past may no longer be a reasonable indicator of the future. Further research will help reduce these uncertainties.

Key hazards to the livestock sector relate not only to climate change trends but also, and importantly, to climate variability and climate extreme events such as heat waves, droughts, floods, cyclones, and wildfires. Increases in inter-annual variability of forage availability is especially a concern for the grazing sector. The increase in frequency, intensity and duration of heat waves also poses a major threat to animal health and human labour where access to mechanization and cooling systems is limited. These climate-related hazards can exacerbate other stressors with negative impacts, especially for people living in poverty. The nature and extent of such impacts are however still largely unknown and warrant further research.

In the face of global warming, and overall harmful impacts as highlighted in this review, the existing suite of adaptation strategies across ecological, socioeconomic, and institutional systems and coping range that have been developed in response to existing weather patterns may not be enough. More transformative climate adaptation may be required. These range from farm management adjustments, technological developments, income-related responses to institutional changes and, in extreme cases, abandonment of livestock keeping (see reviews by Escarcha et al. (2018), Henry et al. (2018), Herrero et al. (2016, 2020), Rivera-Ferre et al. (2016), Salman et al. (2019), Sejian et al. (2015) and Weindl et al. (2015)). Livestock ownership may also be valued as a climate risk-mitigation strategy in some contexts. Indeed, livestock plays multiple roles beyond producing food for the market, especially in smallholder farming systems. For instance, animals are capital assets that can contribute to income diversification. Livestock are also often more resilient than crops to high climate variability.

Adaptation choices and risk management actions across temporal and spatial scales and contexts will need to build on robust methods of designing, implementing and evaluating detailed development pathways. Such pathways, yet to be fully elucidated, must strengthen climate-resilience and limit trade-offs between different actors. Choices and actions will need to account for the widest possible range of potential impacts, including those with low probability but large consequences, because of the large future uncertainties related to hazards, exposure and vulnerabilities, and the large potential consequences for the sector.

## Declaration of competing interest

The authors declare that the research was conducted in the absence of any commercial or financial relationships that could be construed as a potential conflict of interest.
