# Supplementary material for: Impacts of climate change on the livestock food supply chain; a review of the evidence
Source: Glob Food Sec. 2021 Mar;28:100488. doi: 10.1016/j.gfs.2020.100488 (PMC7938222; doi:10.1016/j.gfs.2020.100488)
Supplement: Multimedia component 1 [file mmc1.docx]

# Supplementary Information - Impacts of climate change on the livestock food supply chain; a review of the evidence

C. Godde^1^*, D. Mason-D’Croz^1^, D. Mayberry^1^, P. Thornton^2^, M. Herrero^1^

1. Commonwealth Scientific and Industrial Research Organisation, Agriculture and Food, St Lucia, QLD 4067, Australia.

2. CGIAR Research Programme on Climate Change, Agriculture and Food Security (CCAFS), ILRI, Nairobi, Kenya

*corresponding author: cecile.godde@csiro.au

## 1. Climate Hazards

We briefly describe below some major trends that can impact the production of livestock-sourced food: increases in atmospheric carbon dioxide (eCO_2_) and tropospheric ozone (O_3_) concentrations; changes in both mean and variability of temperature and precipitation; sea level rise and storm surges; and increased risk and frequency of extreme weather events (Figure 1). Impacts of such hazards on the livestock sector are discussed in section 2 of the manuscript.

Since pre-industrial times (1850–⁠1900), atmospheric carbon dioxide (eCO_2)_ concentrations have risen from 290 ppm to 409–⁠415 ppm in 2020 (Scripps Institution of Oceanography, 2020). This is projected to further increase to between 487–⁠541 ppm in 2050 (RCP 4.5–⁠8.5) and 538–⁠936 ppm in 2100, depending on the level of climate mitigation (Riahi et al., 2007; van Vuuren et al., 2011; Van Vuuren et al., 2007). Studies also show that emissions associated with fossil fuel and biomass burning have resulted in an approximate doubling of the mean global tropospheric O_3_ concentration (Ainsworth et al., 2020; Gauss et al., 2006), and further increases are expected in the coming decades under all IPCC emission scenarios, except the most optimistic RCP 2.6 (Eyring et al., 2013).

Of the several uncertainties associated with projections of future climate hazards, an important one is the value of the equilibrium climate sensitivity, the global surface temperature response to a doubling of eCO_2_ concentration. Until recently, this was estimated to lie in the range 1.5–4.5 °C. This sensitivity has increased substantially in the latest generation of climate models, those in the Coupled Model Intercomparison Project phase 6 (CMIP6), with values up to 5.6 °C. This increase has been attributed primarily to stronger positive cloud feedbacks from decreasing extratropical low cloud coverage and albedo in the climate models (Zelinka et al., 2020). Whether this higher sensitivity is plausible is unclear (Forster et al., 2020), but clarification is urgently needed as the ramifications could be very considerable.

It is likely that human activities have caused between 0.8 °C and 1.2 °C of global warming above pre-industrial levels as a result of increased greenhouse gas emissions. This may well reach 1.5 °C between 2030 and 2050 if the current rate of increase continues (IPCC, 2018). Most land regions are projected to experience increases in mean temperature. Extreme hot days (temperature of annual hottest day) in mid-latitudes are projected to warm by about twice the amount of mean global warming; and extreme cold nights (temperature of annual coldest night) in high latitudes warm by up to three times the mean global amount. The number of hot days (10% warmest days) is projected to increase in most land regions, with highest increases in the tropics, while the number of cold days (10% coldest days) is projected to decrease (Hoegh-Guldberg et al., 2018).

Renewable surface water and groundwater resources are projected to decline significantly in most dry subtropical regions, and increase at higher latitudes (Jiménez Cisneros et al., 2014). We expect increases in the frequency, intensity and/or amount of heavy precipitation events in several regions such as central and northern Europe, East Africa and South Asia, and increased probability of drought and precipitation deficits in others such as the Mediterranean and southern Africa (IPCC, 2018). Tropical cyclones may be more damaging with heavier precipitation and increased storm surge due to rising sea levels (Knutson et al., 2020). Global warming of 1.5°C could be expected to increase river flooding hazards in some regions such as Asia, the U.S. and Europe, and land areas susceptible to runoff (Hoegh-Guldberg et al., 2018). Climate change will also increase coastal flood hazards through rising sea levels and increased storm surges (Rahmstorf, 2017). Projections of global mean sea level rise suggest a range of 0.35 to 0.93 m by 2100 for global warming of 2°C, with tens of millions more people exposed to risks associated with saline intrusion and storm surges (Hoegh-Guldberg et al., 2018).

## 2. Vulnerability

### 2.1. Demographic, social and economic drivers

A growing human population, together with increasing incomes and shifts in dietary preferences, predominantly in low- and middle-income countries are driving continued growth in demand for livestock products and resources needed to produce them, including land and water (FAOSTAT, 2020; Herrero et al., 2018). Bovine, sheep and goat meat and milk production have more than doubled since the 1960s, mainly driven by human population growth rather than increased per capita consumption. Over the same period, poultry meat, egg, and pork production have increased globally by a factor of 14.2, 5.5 and 4.9 respectively, driven by population growth, but also by shifting dietary preferences and rapidly increasing incomes in emerging economies. All regions showed production increases for poultry and pork commodities. The largest per capita consumption increase has been estimated for the poultry sector, with global average per capita supply of poultry meat increasing from 2.88 to 14.99 kg/capita/year between 1961 and 2013, and all regions showing positive trends. Potential enforced production systems transitions due to changes in land suitability will affect the food production landscape (Thornton et al., 2019). Large-scale demand for plant-based diets and production of synthetic livestock products may also alter livestock production systems dynamics (Springmann et al., 2018). The development of such food alternatives will be influenced by changing social norms and opportunity-costs of different food production systems under climate change and other global trends.

Human migration, partly induced by climate change, can also increase local pressures on scarce natural resources (FAO, 2018b). Forcibly displaced people are in large majority found in LMICs (85%), in countries or territories currently affected by acute food insecurity and malnutrition (UNHCR, 2020). This increased competition for resources may result in land scarcity for livestock production, land fragmentation and degradation.

Competition between crop as livestock feed and crop for direct human consumption or biofuel production are also increasing (Muscat et al., 2020), and shift in the relative proportion of livestock production systems may occur as demand for poultry is rising (FAOSTAT, 2020; Herrero et al., 2018).

Youth is also increasingly less present farming activities and rural environments, which has implications for the agricultural sector’s performance and adaptive capacity. Key challenges to participation in agriculture include the neglect of small-scale agriculture and rural infrastructure and lack of access to knowledge and education, land, financial services, markets and policy dialogues (FAO, 2014; White, 2012). Production challenges due to harsh climates reinforces this trend. However, opportunities exist. Youth unemployment in rural areas is high in most countries and innovations in small-scale farming are emerging, which are attracting the youth, especially in peri-urban areas and in new and changing agrifood market chains (Proctor and Lucchesi, 2012).

Valuing traditional knowledge, learning and practices will also play a key role in climate resilience (Nielsen et al., 2020). Some of these knowledge systems are however being lost through globalization and development patterns (Ford et al., 2020). Socio-economic and environmental changes may also be such that new adaptation strategies also need exploring across ecological, socioeconomic, and institutional systems.

Remote or marginalised regions with lower economic development levels may be more vulnerable to the impacts of climate change (Hallegatte and Rozenberg, 2017). Limited access to markets can limit off-farm labour use, the sale of livestock products and purchases of fertilizer or feed supplements. It can also limit stock transfer which permits more ready adjustment of stocking rate under climate variability. Household and enterprises with limited capital or access to credit will have difficulties adapting to the impacts of climate change on the livestock sector such as increased prices and volatility and additional labour and infrastructure requirements (Godde et al., 2018). Technology availability and access as well as stakeholders’ education level, gender, values and traditions, social support and perception of risks will also influence the ability of the sector to adapt to and cope with climate change (Godde et al., 2018). Shocks, including climate-related shocks, can trap people into poverty, limiting their ability to accumulate assets and affecting, sometimes in irreversible ways, human capital through health or educational impacts (Hallegatte and Rozenberg, 2017). Climate change may also further exacerbate current social exclusion by increasing resource competition and forcing mass migration.

### 2.2. Political and institutional drivers

Political and institutional forces affect the livestock sector’s vulnerability and robust methods of designing, implementing and evaluating climate-resilient development pathways are needed.

In grazing systems, herd mobility and collective resource management have been key strategies to take advantage of the spatial and temporal variation in water and forage availability. However, increasing land privatization in the Global South has resulted in reductions in pastoralists’ mobility and consequently ability to cope with climate variability (Godde et al., 2018; Reid et al., 2014). Many governments and organisations have aimed to sedentarise nomadic and semi-nomadic households for various reasons, including increasing agricultural productivity, improving water quality, facilitating access to health and education, facilitating tax collection and military conscription, and power consolidation. Land and water policies, lack of investment in remote areas, and conversion of rangelands to other land uses (croplands, urban or recreational areas, mining or industry) also strongly constrain mobility (see regional analyses in Reid et al. (2014)). For instance, in Inner Mongolia the culture had adapted to the harsh climate by using mobility, cooperation, and reciprocity strategies as described in Dalintai et al. (2012). However, collectivization between the 1950s and mid-1980s followed by market reforms in the early 1980s affected the tight connections in the grasslands socio-ecological systems and weakened herd mobility. The state-driven nomad sedentarisation projects in China (Hruska et al., 2017) and transitions from communal to semi-commercial land tenures in southern African rangelands (Dube and Pickup, 2001) have also limited adaptive capacity of people in these regions. In Australia, where most grazing land is owned or leased, it is common practice to move cattle from a droughted area to other privately run properties that have adequate pasture, and graze for a fee ("agistment" strategy; McAllister, 2012; McAllister et al., 2006). The shortage of productive land during widespread droughts can however greatly limit the possibility and economic viability of such a mobility strategy.

Central and local governments’ investments in infrastructure, price support schemes, taxes, credits, subsidies, input and output quotas and the health system, will also influence the vulnerability of livestock supply chains. For example, during COVID-19, there has been a drop in revenues of local governments in the horn of Africa from livestock market sales or slaughters. This has jeopardized their ability to provide services such as market management and maintenance, extension services and drought monitoring (Mercy Corps, 2020). Discrimination based on gender, ethnicity, caste, and wealth impedes participation in markets and legal recognition of land and asset ownership and other rights that play key roles in the ability of stakeholders to adapt to climate change. Institutional disregard of traditional knowledge, institutions and customary practices can also weaken the resilience of livestock systems. Rising food safety standards will also affect the viability of some livestock production systems.

Civil conflicts may also disrupt the livestock sector, increasing its vulnerability to climate change (FAO, 2018b; IPCC, 2014). In some cases, these are related to climate change, as reviewed in FAO, IFAD, UNICEF, WFP (2017), although caution should be exercised when drawing such linkages (Selby et al., 2017) For instance, a region-wide drought in northern Mali in 2012, which lead to the death of thousands of livestock and the devastation of pastoralists’ livelihoods, subsequently resulted in the swelling of the ranks of armed rebels, forcing others to steal for survival (Breisinger et al., 2015).

While some of the political and institutional forces may contribute to persistent trends (e.g. continued exclusion of pastoralists from political processes), others may result in dramatic shifts in the near future and greatly alter the ‘vulnerability landscape’. The pace at which governance factors can change has been exemplified by governments’ actions to stop the spread of diseases. For example, during the H5N1 pandemic, millions of poultry were killed. In Vietnam alone, more than 50 million domestic birds were killed due to HPAI infection and control attempts (McLeod et al., 2005). The impacts on total assets were greatest in small scale commercial and backyard producers, with large implications for food security. Some governments provided compensation for culled poultry, although often well below market values (close to 30% of market value in Vietnam), while others did not provide any compensation at all (e.g. Cambodia).

## 3. Details of calculations – estimates in Introduction section, Tables 2 and 3

**Protein production (g/capita/day)**

Livestock production values per country and livestock production systems were from Herrero et al. (2013) for year 2010. Livestock production systems were from Robinson et al. (2011). Commodities nutritional content and edible proportions were from Nelson et al. (2018) and human population numbers from FAO (2020), averaged over the period 2008-2012.

**Proportion of calorie and protein supply from livestock (%)**

The proportion of kilocalorie and protein supply from livestock is based on food supply (kcal/capita/day) and protein supply (g/capita/day) estimates from FAO (2020) New Food Balances database, averaged over the period 2014-2017.

**Value of production (billion USD, producer price) (2008-2012)**

Livestock production values per country and livestock production systems were from Herrero et al. (2013) for year 2010. Livestock production systems were from Robinson et al. (2011). Commodity prices were from FAO (2020) averaged over the period 2008-2012.

**Proportion of agricultural added value from livestock**

Calculated by dividing FAO (2020) Livestock PIN by Agriculture PIN under FAOSTAT element “Net Production Value (constant 2004-2006 1000 I$)”. Estimates were averaged over the period 2008-2012.

**Population employed in Agriculture - ages 15 and older (million people)**

Population employed in Agriculture - ages 15 and older (people) was estimated by multiplying population numbers by year 2018 country-level agriculture employment (ages 15 and older) to total population ratio. The ratio was itself calculated based on The World Bank (2020) country-level year 2018 employment to population ratio (% ages 15 and older), years 2014-16 agriculture employment as % of total employment, and year 2018 population ages 0-14 (% of total population). When country level year 2018 agriculture employment (ages 15 and older) to total population ratio values were not available, the global average value was taken.

**Proportion of livestock biomass consumption from grazing**

Livestock biomass consumption (grazing biomass, grain, stover and occasionnals) per country and livestock production systems were from Herrero et al. (2013) for year 2010. Livestock production systems were from Robinson et al. (2011).

**Proportion of livestock biomass consumption from grain**

Livestock biomass consumption (grazing biomass, grain, stover and occasionnals) per country and livestock production systems were from Herrero et al. (2013) for year 2010. Livestock production systems were from Robinson et al. (2011).

**Ratio of fodder and feeding stuff quantity imported versus exported in countries**

Fodder and feeding stuff imported and exported quantities per country were sourced from FAO (2020) averaged over the period 2014-2018.

## 4. Details of calculations - human population in arid and hyper arid environments and employment in agriculture

Human population numbers in arid and hyper arid environments in low income countries were estimated by summing population numbers per pixel from HYDE 3.2.1 (Goldewijk, 2017) for each livestock production system in arid and hyper arid environments (LGY, LGA, MRY, MIA, MRY, MRA) from Robinson et al (2011) and for each country. Income classes were defined based on World Bank list of economies, June 2020 (The World Bank, 2020b). The percentage of population employed in Agriculture in low income countries was estimated based on The World Bank (2000) years 2014-16 agriculture employment (% of total employment) at country level weighed by countries’ population numbers.

## 3. Herbaceous dynamics and country-level vulnerability indicators

Figure S1: Country-level mean trends of herbaceous dynamics as projected by G-Range by 2050 (Godde et al. 2020) and country-level livestock vulnerability indicators, as developed and defined by Godber and Wall (2014). Vulnerability indicators: (A) sensitivity score, which represents a nation’s nutritional reliance on grazing animal-based food products and level of food security (0–1 = low to high sensitivity), (B) adaptive capacity score, which represents a measure of a nation’s ability to change in response to or cope with changes in climate and food demand (0–1 = low to high adaptive capacity), (C) population growth score, which represents the impact of projected human population growth on vulnerability of livestock-based food production (0–1 = low to high exposure), (D) Overall vulnerability score, estimated based on the sensitivity, population growth and adaptive capacity scores (0–1 = low to high vulnerability). A positive sign in the legend (+) indicates an increase in the vegetation variable by 2050 and a negative sign (-) a decrease. The solid symbol indicates the mean value. The number of data-points (i.e., countries) in each boxplot is as follows: 11 (blue), 21 (yellow), 4 (orange), 48 (red). Within each of the four panels, groups that were found to have means statistically significantly different from all other boxes in pairwise comparison do not share the same letter (a-b) (Tukey HSD test (p-value<0.05)). The outliers are not represented but accounted for in the statistical analysis. For instance, in the fourth panel, the countries projected to experience simultaneously a decrease in mean herbaceous biomass and an increase in inter-annual variability are countries that have on average the highest vulnerability score. Climate scenario: HadGEM2‐ES RCP 8.5 with atmospheric CO_2_ effects enabled. Further details on livestock vulnerability indicators is provided in Godber and Wall (2014). Figure from Godde et al. (2020).

Table S1: Country-level mean trends of herbaceous dynamics as projected by G-Range by 2050 (Godde et al. 2020) and country-level livestock vulnerability indicators, as developed and defined by Godber and Wall (2014). Herbaceous dynamics trends: (a; blue colour) increase in mean herbaceous biomass and decrease in biomass inter-annual variability; (b; yellow) increase in mean herbaceous biomass and biomass inter-annual variability; (c; orange) decrease in mean herbaceous biomass and biomass inter-annual variability; (d; red) decrease in mean herbaceous biomass and increase in biomass inter-annual variability. Vulnerability indicators: sensitivity score, which represents a nation’s nutritional reliance on grazing animal-based food products and level of food security (0–1 = low to high sensitivity); adaptive capacity score, which represents a measure of a nation’s ability to change in response to or cope with changes in climate and food demand (0–1 = low to high adaptive capacity); population growth score, which represents the impact of projected human population growth on vulnerability of livestock-based food production (0–1 = low to high exposure); overall vulnerability score, estimated based on the sensitivity, population growth and adaptive capacity scores (0–1 = low to high vulnerability). Table ordered by decreasing vulnerability score value. Bold indicates upper tenth percentile, italics indicates lower tenth percentiles. See main manuscript for further details about how rangeland area was calculated. Climate scenario: HadGEM2‐ES RCP 8.5 with atmospheric CO_2_ effects enabled. Further details on livestock vulnerability indicators is provided in Godber and Wall (2014). Table from Godde et al. (2020).

| **Country name** | **Herbaceous dynamics trends** | **Rangeland area (Mha)** | **Sensitivity score** | **Adaptive capacity score** | **Population growth score** | **Vulnerability score** |
| --- | --- | --- | --- | --- | --- | --- |
| Eritrea | d | 4.5 | **0.89** | 0.17 | 0.35 | **1** |
| Sudan | d | **63.2** | **0.89** | 0.17 | 0.35 | **1** |
| Burundi | a | ***0.09*** | **0.75** | ***0.03*** | 0.28 | **0.96** |
| Kenya | d | 18.1 | **0.7** | 0.12 | 0.38 | **0.94** |
| Chad | d | 38.0 | 0.51 | ***0.02*** | **0.44** | **0.91** |
| Zambia | b | 24.0 | 0.57 | ***0.01*** | 0.35 | **0.9** |
| Central African Republic | d | 0.8 | 0.61 | ***0*** | 0.28 | **0.89** |
| Mongolia | d | **80.5** | **1** | 0.26 | 0.15 | **0.89** |
| Uganda | d | 1.3 | 0.49 | 0.08 | **0.45** | **0.87** |
| Ethiopia | b | 9.6 | 0.61 | 0.14 | 0.38 | 0.86 |
| Niger | d | 17.5 | 0.45 | 0.09 | **0.48** | 0.86 |
| Swaziland | d | 0.3 | **0.64** | ***0.01*** | 0.23 | 0.86 |
| United Republic of Tanzania | d | 31.7 | 0.58 | 0.13 | 0.39 | 0.86 |
| Angola | d | **42.0** | 0.43 | ***0.04*** | **0.44** | 0.84 |
| Botswana | d | 17.6 | **0.64** | 0.13 | 0.27 | 0.82 |
| Namibia | d | 23.4 | **0.66** | 0.21 | 0.34 | 0.82 |
| Pakistan | b | 0.4 | **0.71** | 0.23 | 0.31 | 0.82 |
| Mali | d | 25.5 | 0.4 | 0.05 | **0.41** | 0.81 |
| Mozambique | d | 34.6 | 0.42 | ***0.03*** | 0.38 | 0.81 |
| Yemen | d | 10.0 | 0.5 | 0.22 | **0.49** | 0.81 |
| Madagascar | d | 18.3 | 0.55 | 0.24 | **0.42** | 0.79 |
| Mauritania | d | 8.8 | 0.48 | 0.14 | 0.39 | 0.79 |
| Bolivia | d | 12.8 | 0.63 | 0.24 | 0.28 | 0.75 |
| Burkina Faso | d | 1.6 | 0.37 | 0.1 | 0.39 | 0.75 |
| Colombia | b | 17.0 | **0.75** | 0.34 | 0.24 | 0.74 |
| Rwanda | a | ***0.10*** | 0.46 | 0.1 | 0.28 | 0.73 |
| Turkmenistan | d | 20.6 | 0.63 | 0.22 | 0.23 | 0.73 |
| Cameroon | d | 0.6 | 0.33 | 0.05 | 0.34 | 0.71 |
| Senegal | d | 3.2 | 0.38 | 0.15 | 0.38 | 0.71 |
| Guinea | d | 3.0 | 0.28 | 0.08 | 0.38 | 0.69 |
| Lesotho | d | 1.2 | 0.38 | ***0*** | 0.2 | 0.68 |
| Malawi | b | 0.8 | 0.31 | 0.08 | 0.32 | 0.68 |
| Guinea Bissau | d | 0.6 | 0.26 | ***0*** | 0.28 | 0.66 |
| Tajikistan | b | 1.2 | 0.6 | 0.25 | 0.18 | 0.66 |
| Uzbekistan | d | 13.5 | 0.56 | 0.26 | 0.23 | 0.66 |
| Ivory Coast | d | 8.5 | 0.28 | 0.09 | 0.32 | 0.65 |
| Kyrgyzstan | c | 6.3 | **0.64** | 0.27 | 0.14 | 0.65 |
| Guatemala | b | ***0.09*** | 0.44 | 0.3 | 0.34 | 0.63 |
| Togo | d | ***0.10*** | 0.25 | 0.12 | 0.35 | 0.63 |
| Ecuador | b | 1.8 | 0.58 | 0.35 | 0.24 | 0.62 |
| India | c | 0.5 | 0.49 | 0.28 | 0.25 | 0.62 |
| Gambia | d | 0.2 | ***0.17*** | 0.14 | **0.41** | 0.61 |
| Paraguay | d | 13.8 | 0.46 | 0.31 | 0.3 | 0.61 |
| Nigeria | d | 10.5 | ***0.16*** | 0.06 | 0.34 | 0.6 |
| South Africa | b | 38.9 | 0.28 | 0.09 | 0.25 | 0.6 |
| Argentina | b | **69.4** | 0.62 | 0.37 | 0.15 | 0.58 |
| Azerbaijan | a | 0.5 | 0.51 | 0.29 | 0.17 | 0.57 |
| Jordan | d | 0.134 | 0.25 | 0.33 | **0.45** | 0.56 |
| Democratic Republic of the Congo | b | 3.7 | ***0.11*** | 0.12 | 0.37 | 0.55 |
| Syria | b | 2.8 | 0.32 | 0.36 | 0.35 | 0.53 |
| Georgia | c | 0.3 | 0.63 | 0.33 | ***0*** | 0.51 |
| Venezuela | d | 9.6 | 0.35 | 0.35 | 0.27 | 0.5 |
| Saudi Arabia | d | 0.6 | ***0.23*** | 0.35 | 0.38 | 0.49 |
| Algeria | d | 12.8 | 0.34 | 0.32 | 0.24 | 0.48 |
| Kazakhstan | b | **136.0** | 0.51 | 0.27 | ***0*** | 0.48 |
| Ghana | d | 6.2 | ***0.1*** | 0.22 | 0.35 | 0.47 |
| Peru | b | 10.2 | 0.36 | 0.34 | 0.21 | 0.47 |
| Brazil | b | **91.1** | 0.44 | 0.41 | 0.18 | 0.46 |
| Iran | d | 38.2 | 0.34 | 0.33 | 0.21 | 0.46 |
| Armenia | a | 0.2 | 0.52 | 0.33 | ***0*** | 0.44 |
| Egypt | d | ***0.09*** | 0.25 | 0.33 | 0.25 | 0.44 |
| Morocco | b | 8.2 | 0.26 | 0.31 | 0.18 | 0.41 |
| Libya | d | 8.3 | ***0.19*** | 0.34 | 0.27 | 0.4 |
| Mexico | d | 36.6 | 0.31 | 0.4 | 0.21 | 0.4 |
| Russia | d | 15.8 | 0.42 | 0.32 | ***0*** | 0.39 |
| Turkey | b | 3.0 | 0.24 | 0.36 | 0.21 | 0.38 |
| Indonesia | d | ***0.125*** | ***0.18*** | 0.29 | 0.18 | 0.37 |
| Bosnia and Herzegovina | a | ***0.08*** | 0.4 | 0.35 | ***0*** | 0.36 |
| Guyana | a | 0.6 | 0.3 | 0.28 | ***0.03*** | 0.36 |
| Macedonia | c | 0.15 | 0.34 | 0.35 | ***0.06*** | 0.36 |
| Tunisia | a | 0.7 | ***0.22*** | 0.35 | 0.18 | 0.36 |
| Uruguay | b | 13.1 | 0.43 | 0.45 | ***0.06*** | 0.35 |
| Chile | b | 4.9 | 0.34 | **0.57** | 0.18 | 0.29 |
| Croatia | a | 0.3 | 0.29 | 0.4 | ***0*** | 0.26 |
| China | b | **177.3** | 0.26 | 0.51 | 0.11 | 0.24 |
| Australia | d | **195.1** | 0.41 | **0.76** | 0.18 | ***0.22*** |
| Ireland | d | ***0.08*** | 0.38 | **0.71** | 0.17 | ***0.22*** |
| Italy | d | 0.8 | 0.29 | 0.54 | ***0.04*** | ***0.19*** |
| New Zealand | b | 7.1 | 0.44 | **0.83** | 0.17 | ***0.19*** |
| Spain | a | 0.3 | ***0.21*** | **0.55** | 0.13 | ***0.19*** |
| France | a | 2.6 | 0.41 | **0.7** | 0.07 | ***0.18*** |
| Switzerland | a | ***0.07*** | 0.42 | **0.85** | 0.11 | ***0.12*** |
| Canada | d | 0.2 | 0.26 | **0.79** | 0.14 | ***0.08*** |
| United States of America | d | **142.7** | 0.35 | **1** | 0.15 | ***0.01*** |

## 5. References

Ainsworth, E.A., Lemonnier, P., Wedow, J.M., 2020. The influence of rising tropospheric carbon dioxide and ozone on plant productivity. Plant Biol. 22, 5–11. https://doi.org/10.1111/plb.12973

Breisinger, O., Ecker, Tan, J.F.T., 2015. Conflict and food insecurity: How do we break the links?, in: Global Food Policy Report 2014–2015. IFPRI, Washington, pp. 51–59.

Eyring, V., Arblaster, J.M., Cionni, I., Sedláček, J., Perlwitz, J., Young, P.J., Bekki, S., Bergmann, D., Cameron-Smith, P., Collins, W.J., Faluvegi, G., Gottschaldt, K.D., Horowitz, L.W., Kinnison, D.E., Lamarque, J.F., Marsh, D.R., Saint-Martin, D., Shindell, D.T., Sudo, K., Szopa, S., Watanabe, S., 2013. Long-term ozone changes and associated climate impacts in CMIP5 simulations. J. Geophys. Res. Atmos. 118, 5029–5060. https://doi.org/10.1002/jgrd.50316

FAO, IFAD, UNICEF, WFP, W., 2017. The State of Food Security and Nutrition in the World 2017. Building resilience for peace and food security. Rome.

FAO, 2018. The Impact of disasters and crises on agriculture and Food Security. Rome.

FAOSTAT, 2020. FAOSTAT [WWW Document]. URL http://www.fao.org/faostat/en/ (accessed 4.24.20).

Forster, P.M., Maycock, A.C., McKenna, C.M., Smith, C.J., 2020. Latest climate models confirm need for urgent mitigation. Nat. Clim. Chang. 10, 7–10. https://doi.org/10.1038/s41558-019-0660-0

Gauss, M., Myhre, G., Isaksen, I.S.A., Grewe, V., Pitari, G., Wild, O., Collins, W.J., Dentener, F.J., Ellingsen, K., Gohar, L.K., Hauglustaine, D.A., Iachetti, D., Lamarque, J.F., Mancini, E., Mickley, L.J., Prather, M.J., Pyle, J.A., Sanderson, M.G., Shine, K.P., Stevenson, D.S., Sudo, K., Szopa, S., Zeng, G., 2006. Radiative forcing since preindustrial times due to ozone change in the troposphere and the lower stratosphere. Atmos. Chem. Phys. 6, 575–599. https://doi.org/10.5194/acp-6-575-2006

Godber, O.F., Wall, R., 2014. Livestock and food security: Vulnerability to population growth and climate change. Glob. Chang. Biol. 20, 3092–3102. https://doi.org/10.1111/gcb.12589

Godde, C., Garnett, T., Thornton, P., Ash, A., Herrero, M., 2018. Grazing systems expansion and intensification: Drivers, dynamics, and trade-offs. Glob. Food Sec. 16, 93–105. https://doi.org/10.1016/j.gfs.2017.11.003

Godde, C.M., Boone, R., Ash, A.J., Waha, K., Sloat, L., Thornton, P.K., Herrero, M., 2020. Global rangeland production systems and livelihoods at threat under climate change and variability. Environ. Res. Lett. 15, 44021. https://doi.org/10.1088/1748-9326/ab7395

Goldewijk, Klein, Dr. ir. C.G.M. (Utrecht University) (2017): *Anthropogenic land-use estimates for the Holocene; HYDE 3.2*. DANS. https://doi.org/10.17026/dans-25g-gez3

Hallegatte, S., Rozenberg, J., 2017. Climate change through a poverty lens. Nat. Clim. Chang. 7, 250–256. https://doi.org/10.1038/nclimate3253

Herrero, M., Mason-D’Croz, D., Godde, C.M., Palmer, J., Thornton, P.K., Gill, M., 2018. Livestock, land and the environmental limits of animal source-food consumption, in: CGIAR Science Forum 2018. CGIAR, Stellenbosch, South Africa, pp. 1–39.

Hoegh-Guldberg, O., Jacob, D., Taylor, M., Bindi, M., Brown, S., Camilloni, I., Diedhiou, A., Djalante, R., Ebi, K.L., Engelbrecht, F., Guiot, J., Hijioka, Y., Mehrotra, S., Payne, A., Seneviratne, S.I., Thomas, A., Warren, R., Zho, G., 2018. Impacts of 1.5oC Global Warming on Natural and Human Systems, in: Masson-Delmotte, V., Zhai, P., Pörtner, H.-O., Roberts, D., Skea, J., Shukla, P.R., Pirani, A., Moufouma-Okia, W., Péan, C., Pidcock, R., Connors, S., Matthews, J.B.R., Chen, Y., Zhou, X., Gomis, M.I., Lonnoy, E., Maycock, T., Tignor, M., Waterfield, T. (Eds.), Global Warming of 1.5°C. An IPCC Special Report on the Impacts of Global Warming of 1.5°C above Pre-Industrial Levels and Related Global Greenhouse Gas Emission Pathways, in the Context of Strengthening the Global Response to the Threat of Climate Change,. pp. 175–311.

IPCC, 2018. Summary for Policymakers, in: Masson-Delmotte, V., P.Z., Pörtner, H.-O., Roberts, D., Skea, J., Shukla, P.R., Pirani, A., Moufouma-Okia, W., Péan, C., Pidcock, R., Connors, S., Matthews, J.B.R., Chen, Y., Zhou, X., Gomis, M.I., Lonnoy, E., Maycock, T., Tignor, M., Waterfield, T. (Eds.), Global Warming of 1.5°C. An IPCC Special Report on the Impacts of Global Warming of 1.5°C above Pre-Industrial Levels and Related Global Greenhouse Gas Emission Pathways, in the Context of Strengthening the Global Response to the Threat of Climate Change,. https://doi.org/10.1016/j.oneear.2019.10.025

IPCC, 2014. Climate Change 2014 Part A: Global and Sectoral Aspects, Climate Change 2014: Impacts, Adaptation, and Vulnerability. Part A: Global and Sectoral Aspects. Contribution of Working Group II to the Fifth Assessment Report of the Intergovernmental Panel on Climate Change.

Jiménez Cisneros, B.E., Oki, T., Arnell, N.W., Benito, G., Cogley, J.G., Döll, P., Jiang, T., Mwakalila, S.S., 2014. Freshwater Resources, in: Field, C.B., Barros, V.R., Dokken, D.J., Mach, K.J., Mastrandrea, M.D., Bilir, T.E., Chatterjee, M., Ebi, K.L., Estrada, Y.O., Genova, R.C., Girma, B., Kissel, E.S., Levy, A.N., MacCracken, S., Mastrandrea, P.R., White, L.L. (Eds.), Climate Change 2014: Impacts, Adaptation, and Vulnerability. Part A: Global and Sectoral Aspects. Contribution of Working Group II to the Fifth Assessment Report of the Intergovernmental Panel on Climate Change. Cambridge University Press, Cambridge, United Kingdom and New York, NY, USA, pp. 229–269.

Knutson, T., Camargo, S.J., Chan, J.C.L., Emanuel, K., Ho, C.H., Kossin, J., Mohapatra, M., Satoh, M., Sugi, M., Walsh, K., Wu, L., 2020. Tropical cyclones and climate change assessment. Bull. Am. Meteorol. Soc. 101, E303–E322. https://doi.org/10.1175/BAMS-D-18-0189.1

McLeod, A., Morgan, N., Prakash, A., Hinrichs, J., 2005. Economic and social impacts of avian influenza. Rome.

Mercy Corps, 2020. COVID-19 and livestocj market systems: The impact of COVID-19 on livestock-based economies in the Horn of Africa. Portland.

Muscat, A., de Olde, E.M., de Boer, I.J.M., Ripoll-Bosch, R., 2020. The battle for biomass: A systematic review of food-feed-fuel competition. Glob. Food Sec. 25, 100330. https://doi.org/10.1016/j.gfs.2019.100330

Rahmstorf, S., 2017. Rising hazard of storm-surge flooding. Proc. Natl. Acad. Sci. U. S. A. 114, 11806–11808. https://doi.org/10.1073/pnas.1715895114

Reid, R.S., Fernandez-Gimenez, M.E., Galvin, K.A., 2014. Dynamics and resilience of rangelands and pastoral peoples around the globe. Annu. Rev. Environ. Resour. 39, 217–42. https://doi.org/10.1146/annurev-environ-020713-163329

Riahi, K., Grübler, A., Nakicenovic, N., 2007. Scenarios of long-term socio-economic and environmental development under climate stabilization. Technol. Forecast. Soc. Change 74, 887–935. https://doi.org/10.1016/j.techfore.2006.05.026

Robinson TP, Thornton PK, Franceschini G, Kruska RL, Chiozza F, Notenbaert A, Cecchi G, Herrero M, Epprecht M, Fritz S, You L, Conchedda G & See L (2011) Global livestock production systems. Rome: Food and Agriculture Organization of the United Nations (FAO) and International Livestock Research Institute (ILRI). pp. 152.

Scripps Institution of Oceanography, 2020. The Keeling Curve [WWW Document]. URL https://sioweb.ucsd.edu/programs/keelingcurve/ (accessed 10.2.20).

Selby, J., Dahi, O.S., Fröhlich, C., Hulme, M., 2017. Climate change and the Syrian civil war revisited. Polit. Geogr. 60, 232–244. https://doi.org/10.1016/j.polgeo.2017.05.007

Springmann, M., Clark, M., Mason-D’Croz, D., Wiebe, K., Bodirsky, B.L., Lassaletta, L., de Vries, W., Vermeulen, S.J., Herrero, M., Carlson, K.M., Jonell, M., Troell, M., DeClerck, F., Gordon, L.J., Zurayk, R., Scarborough, P., Rayner, M., Loken, B., Fanzo, J., Godfray, H.C.J., Tilman, D., Rockström, J., Willett, W., 2018. Options for keeping the food system within environmental limits. Nature 562, 519–525. https://doi.org/10.1038/s41586-018-0594-0

The World Bank, 2020. World Bank list of economies [WWW Document]. URL https://datahelpdesk.worldbank.org/knowledgebase/articles/906519-world-bank-country-and-lending-groups (accessed 10.10.20).

The World Bank, 2020. World Development Indicators [WWW Document]. URL https://datacatalog.worldbank.org/dataset/world-development-indicators (accessed 30.06.20).

Thornton, P., Herrero, M., Boone, R., 2019. Altered grazing systems: Pastoralism to conventional agriculture, in: Gibson, D.J., Newman, J.A. (Eds.), Grasslands and Climate Change. Cambridge University Press, pp. 253–273.

UNHCR, 2020. Global Trends: Forced Displacements in 2019. Copenhagen.

Van Vuuren, D.P., Den Elzen, M.G.J.J., Lucas, P.L., Eickhout, B., Strengers, B.J., Van Ruijven, B., Wonink, S., Van Houdt, R., 2007. Stabilizing greenhouse gas concentrations at low levels: An assessment of reduction strategies and costs. Clim. Change 81, 119–159. https://doi.org/10.1007/s10584-006-9172-9

van Vuuren, D.P., Edmonds, J., Kainuma, M., Riahi, K., Thomson, A., Hibbard, K., Hurtt, G.C., Kram, T., Krey, V., Lamarque, J.F., Masui, T., Meinshausen, M., Nakicenovic, N., Smith, S.J., Rose, S.K., 2011. The representative concentration pathways: an overview. Clim. Change 109, 5–31. https://doi.org/10.1007/s10584-011-0148-z

Zelinka, M.D., Myers, T.A., McCoy, D.T., Po-Chedley, S., Caldwell, P.M., Ceppi, P., Klein, S.A., Taylor, K.E., 2020. Causes of Higher Climate Sensitivity in CMIP6 Models. Geophys. Res. Lett. 47, e2019GL085782. https://doi.org/10.1029/2019GL085782
